# Change in inflammatory parameters in prefrail and frail persons obtaining physical training and nutritional support provided by lay volunteers: A randomized controlled trial

**DOI:** 10.1371/journal.pone.0185879

**Published:** 2017-10-12

**Authors:** Sandra Haider, Igor Grabovac, Eva Winzer, Ali Kapan, Karin Emmi Schindler, Christian Lackinger, Sylvia Titze, Thomas Ernst Dorner

**Affiliations:** 1 Department of Social and Preventive Medicine, Centre for Public Health, Medical University of Vienna; Austria; 2 Department of Internal Medicine III, Division of Endocrinology and Metabolism, Medical University of Vienna; Austria; 3 Department for Health Promotion and Prevention, SPORTUNION; Vienna, Austria; 4 Institute of Sport Science, University of Graz; Graz, Austria; TNO, NETHERLANDS

## Abstract

The aim of the study was to compare the effects of home visits with physical training and nutritional support on inflammatory parameters to home visits with social support alone within a randomized controlled trial. Prefrail and frail persons received home visits from lay volunteers twice a week for 12 weeks. Participants in the physical training and nutritional intervention group (PTN, n = 35) conducted two sets of six strength exercises and received nutritional support. The social support group (SoSu, n = 23) received visits only. TNF-α, IL-6, CRP, and total leukocyte count were assessed at baseline and after 12 weeks. Changes over time within groups were analyzed with paired t-tests; differences between groups were analyzed with ANCOVA for repeated measurements. In the PTN group, IL-6 and CRP remained stable, whereas in the SoSu group, IL-6 increased significantly from a median value of 2.6 pg/l (min–max = 2.0–10.2) to 3.0 pg/l (min–max = 2.0–20.8), and CRP rose from 0.2 mg/dl (min–max = 0.1–0.9) to 0.3 mg/dl (min–max = 0.1–3.0) after 12 weeks. In CRP, a significant difference between groups was found. TNF-α and total leukocyte count did not change in either the PTN group or the SoSu group. Persons showing an increase in physical performance (OR 4.54; 95% CI = 1.33–15.45) were more likely to have constant or decreased IL-6 values than persons who showed no improvement. In conclusion, in non-robust older adults, a physical training and nutritional support program provided by lay volunteers can delay a further increase in some inflammatory parameters.

## Introduction

Cross-sectional studies have shown that the geriatric syndrome of frailty is linked to increased inflammatory parameters [1, 2]. Accordingly, tumor necrosis factor alpha (TNF-α) [3, 4], interleukin 6 (IL-6) [4–8], C-reactive protein (CRP) [3, 4, 8–10], and total leukocyte count [4, 5, 8] are higher in frail persons than in robust persons. As lower muscle mass and muscle strength, poor physical performance, and decreased functional abilities are associated with higher immediate inflammatory parameters in older adults, these factors might influence chronic inflammation [11, 12]. In very old and frail individuals, however, muscle mass is not related to TNF-α, IL-6, and CRP [13, 14], and physical performance has only a limited association with IL-6 and CRP [15]. It has also been shown that chronic inflammation in older persons can result in a future decrease in muscle mass [16, 17], higher incidence of physical disabilities [18], diabetes mellitus [19], hypertension and myocardial infarction [15], ischemic stroke and transient attacks [20], as well as all-cause mortality [21].

Cross-sectional studies have demonstrated that physical activity is inversely associated with chronic inflammatory parameters in older persons [22, 23], with biochemical changes in muscles or in body composition (e.g. a decline in fat mass) being the likely cause [12, 24]. Nonetheless, the exact mechanism is not currently known [12, 24, 25], and the effects of a physical and nutritional intervention on inflammatory parameters in older persons have only been examined in a few intervention studies, with controversial results. For example, a study in obese frail persons found that a 12-week program had no effect on TNF- α and IL-6 levels [26]. In another study in obese older persons with knee osteoarthritis, where frailty was not an inclusion criterion, a decrease in IL-6 and CRP but not in TNF-α was shown after 6 months. In addition, a decrease in IL-6 with no change in CRP was found in adults at risk of disability [27]. This decline in IL-6 is also in line with a study of persons older than 60 years participating in a strength training program [28].

Besides physical activity, body composition and some macro- and micro-nutrients influence the inflammatory parameters. In overweight and obese individuals, it has been convincingly demonstrated that weight loss reduces CRP and IL-6 [29]. The reduction in fat mass, which decreases IL-6 and in turn lowers CRP, might be the underlying reason for this [30]. These effects only apply to obese persons, and findings in prefrail and frail subjects are broadly missing. Polyphenols, which occur in vegetables and fruits [31–33], and the supplementation of oleic and alpha-linolenic acid, have also been shown to have anti-inflammatory effects [29]. The influences of saturated and trans-fatty acids, cholesterol, soy intake, vitamin D, and whey supplements are conflicting [29, 34], while most studies have shown that fish oil supplementation does not have any effect [29].

In a previously published paper, we looked at the effects of the intervention on handgrip strength, which was our main outcome parameter. We demonstrated that home visits with physical training and nutritional support provided by lay volunteers can increase handgrip strength significantly, with no significant difference to home visits with social support alone [35]. Fat mass and appendicular muscle mass were neither affected by home visits with physical training and nutritional intervention nor by home visits with social support alone. Up to now, it is not known whether such an intervention can also affect inflammatory parameters. Therefore, the aim of the present paper was to examine the effects of home visits with physical training and nutritional support carried out by lay volunteers on inflammatory parameters, and to compare them to the effects of home visits with social support alone. In addition, we aimed to examine the association between inflammatory parameters and baseline values of body composition, handgrip strength, and physical performance. A further purpose was to look at parameters associated with constant or decreased inflammatory parameters within 12 weeks.

## Materials and methods

The study includes data from a randomized controlled trial that took place in Vienna from September 2013 to July 2015, Austria. Recruitment was undertaken between 09/2013 and 10/2014; last follow-up assessment was conducted in 02/2015. The study complies with the Declaration of Helsinki [36]. Ethical approval was given by the ethical committee of the Medical University of Vienna (ref: 1416/2013; date: 27.08.2013), and written informed consent was obtained from all participants. The protocol was registered at ClinicalTrials.gov (identifier: NCT01991639). All ongoing and related trials were also registered. The study protocol, also describing the sample size calculation, which is based on the main outcome of handgrip strength, has previously been published [37]. Based on this calculation (two-sided t-test, significance level = 0.05, power = 80%, differences between the groups: 2 kg with a standard deviation of 3 kg), we aimed to include 40 participants in each group. Randomization was done with the help of the Randomizer for Clinical Trials 1.8.1 [38]. In this paper, we focus on changes in inflammatory parameters, which was prospectively defined as a secondary outcome.

### Participants

#### Prefrail or frail individuals

The inclusion and exclusion criteria have previously been described in the study protocol [37]. Community-dwelling prefrail or frail individuals according to the Survey of Health, Ageing and Retirement in Europe (SHARE-FI) were included [39]. The SHARE-FI includes the items of “exhaustion”, “loss of appetite”, “weakness”, “slowness”, and “low physical activity”. The ratings of the five items are summarized to form a discrete factor score (DFS). As at least “pre-frailty” was an inclusion criterion of this study, women with a DFS of >0.315 and men with a DFS of >1.212 were included [37]. In addition, persons at least at risk of malnutrition according to the Mini Nutritional Assessment Short-Form (MNA-SF ≤11 points) were included [40]. Further inclusion criteria were: resident in Vienna and older than 65 years [37]. Individuals with: insulin-treated diabetes mellitus, chronic obstructive pulmonary disease stage III or IV, chronic kidney insufficiency with protein restriction, or chemo- or radio-therapy during the study period were excluded. Persons with insufficient German language skills, impaired cognitive function according to the Mini-Mental State Examination (MMSE ≤17 points) [41], or any contraindication for physical training were also excluded. In addition to these criteria, we also excluded individuals with any acute infection at baseline from the analyses of this paper. These individuals met the following two criteria: 1) an acute infection was defined as an illness with a rapid onset, leading to a substantial decrease in health during the last month leading up to baseline [42]; and 2) excluded persons had high inflammatory parameters at baseline, which then decreased strongly during the study period. At the very beginning of the study, the study team tried to recruit patients at three different hospitals in internal medicine wards. As this recruitment mode was not successful, patients were recruited through two editorial features, one in the local newspaper and one on television, describing the content of the study. Inclusion criteria were then checked by the study team.

#### Buddies

Prefrail or frail individuals were visited by a lay-volunteer called a “buddy”. These buddies were recruited through notices in newspapers and had to be older than 50 years. Prior to inclusion, the buddies received detailed information about the study during an information evening. Furthermore, their intention to take part in the study was checked within a structured interview by a psychologist.

### Intervention

#### Training of the buddies

Before the home visits were started, buddies received four training sessions, each with a mean duration of 3 hours. These training sessions were performed by a multidisciplinary team consisting of sports and nutritional scientists, physiotherapists, dieticians, psychologists, and medical doctors. Discussed themes included: the ageing process, frailty, malnutrition, and physical activity in older adults. In addition, buddies were trained in the implementation of the physical training and nutritional intervention.

The study consisted of two study groups:

- Physical training and nutritional intervention (PTN) group: As sarcopenia and malnutrition contribute to the frailty syndrome, physical training (especially strength training) and nutritional intervention play a relevant role in tackling frailty [43, 44]. Therefore, participants were asked to perform 6−10 strength exercises with 1−3 sets and 18−20 repetitions in circuit form at least twice a week [45]. In addition, a diet sufficient in energy, protein, and micronutrients was required [46, 47].
  Based on this knowledge, the PTN group received physical training and nutritional support twice a week. In detail, participants performed a warm-up session with mobilization exercises, followed by six strength exercises, which were performed in circuit form. The following exercises were conducted: 1) mini squats in front of a chair; 2) chest presses against elastic resistance; 3) an exercise for the abdominal muscles performed in sitting position; 4) hip extensions in standing position; 5) reverse butterfly; and 6) shoulder presses against elastic resistance. This circuit was conducted twice with 15 repetitions until muscular exhaustion. If the participants could not perform an exercise due to physical limitations, the exercise was skipped.
  During these home visits, three main nutritional messages were also discussed, which were aimed at improving fluid, protein, and energy intake. Fruit, vegetable, and cereal intake was also addressed. For this purpose, buddies were provided with a portfolio describing the recommended intake, the relevance, as well as implementation possibilities. Participants were also provided with the Healthy for Life Plate [37], a modification of the Healthy Eating Plate [48], which shows the differences between recommended and actual food intake. Participants were also provided with a recipe book, which included ideas for protein-rich dishes.
- Social support (SoSu) group: Participants in the SoSu group received home visits without physical training and nutritional support. They were allowed to spend their time as they wanted (e.g. they were free to talk with the buddy about everyday things or they could conduct cognitive training).

### Measurements

The following measurements were conducted at baseline and after 12 weeks:

#### Inflammatory parameters

Blood was taken by a mobile laboratory at the home of the frail participants. The time of the blood draw was not standardized. However, it was ensured that blood was not taken within 8 hours of exercise training. The following inflammatory parameters were obtained: TNF-α (pg/ml) and IL-6 (pg/ml) using chemiluminescent immunoassay, CRP (mg/dl) using immunoturbidimetry, and total leukocyte count (g/l). In this paper, parameters are shown as metric variables. In addition, TNF-α, IL-6, and CRP were dichotomized as “normal” or “elevated” values according to the cut-off values for very old community-dwelling individuals (women = TNF-α: 3.0 pg/ml; IL-6: 1.4 pg/ml; CRP: 0.1 mg/dl; men = TNF-α: 3.3 pg/ml; IL-6: 1.8 pg/ml; CRP: 0.1 mg/dl) [13]. Total leukocyte counts were also dichotomized [49].

#### Body mass index (BMI)

In order to assess BMI (kg/m^2^), body weight was determined with a Marsden MS-4203® calibrated scale (Rotherham, UK) and body height was measured with a tape.

#### Fat mass (FM), appendicular skeletal muscle mass (ASMM), and phase angle

These three parameters were assessed with a phase-sensitive bioelectrical impedance analysis (BIA) device (BIA 2000-S, Data Input®, Darmstadt, Germany) in lying position with four electrodes [50]. Within this method, an alternating current is sent through the body, measuring body resistance (R) and reactance (X_c_). FM in kg was calculated by subtracting fat-free mass from body weight. Fat-free mass was calculated with the validated Geneva formula [−4.104 + (0.518 × height^2^/resistance) + (0.231 × weight) + (0.130 × reactance) + (4.229 × sex: men = 1, women = 0)] [51], as it is known to be the best available method in individuals aged ≥65 years [52].

ASMM in kg was calculated using the validated formula of Sergi et al. [–3.964 +(0.227 × height^2^/R) + (0.095 × weight) + (1.384 × sex) +(0.064 × X_c_)] [53]. To obtain relative values in kg/m^2^, the absolute values were divided by body height in meters, resulting in the fat mass index (FMI) and appendicular skeletal muscle mass index (ASMMI). In addition, phase angle [R/ X_c_ ×180°/ π] was taken from the BIA device.

#### Handgrip strength

Maximum handgrip strength in kg was measured using a JAMAR® handheld dynamometer (Lafayette, Louisiana), for both hands, three times each. For this purpose, participants were seated with their arms bent at the elbow, and they were required to grip with maximal effort [54]. The maximum value achieved was noted. In addition, relative handgrip strength in kg/m^2^ was calculated by dividing absolute values by body height.

#### Physical performance

Physical performance was assessed with the Short Physical Performance Battery (SPPB) [55]. In this test, balance, gait speed, and five timed chair stands are assessed. The lowest reachable value is 0 points; the best value is 12 points.

#### Comorbidities

The information about existing comorbidities is based on self-reports, and was verified with the daily medication records. The Charlson comorbidity index (CI) was calculated by summing the weighted comorbidities, and adding a score for the age [56]. Participants were divided into individuals with “mild comorbidities” (CI <2), “moderate comorbidities” (2≤ CI ≤4), and “severe comorbidities” (CI ≥5).

#### Medication

The information about currently taken medication is based on the delivered drug packages. The name of the patient and the daily dose were noted. As drugs with anti-inflammatory effects influence inflammatory parameters, individuals were classified into persons taking medication with anti-inflammatory effects and persons who did not. Drugs with the following chemical agents were classified as anti-inflammatory drugs: ciclesonide, dexibuprofen, diclofenac, fluticasone-propionate, ibuprofen, lornoxicam, mefenamic acid, meloxicam, naproxen, and prednisone. The total number of drugs taken and the intake of anti-inflammatory drugs (yes, no) are presented in this paper.

#### Adherence

The information about the adherence to the intervention was noted in documentation forms by the buddies. Frequency and duration of the home visits, numbers of circuits, exercises, and repetitions were noted. Furthermore, the content and the numbers of nutritional interventions were recorded. The number of training sessions between home visits was also noted.

### Statistical methods

The normal distribution of the included data was checked with histograms and box plots. Continuous normally distributed data are presented as the mean (standard deviation) and skewed data as the median (minimum–maximum). Categorical variables are shown as frequencies (percentages). In order to test the differences between the PTN and the SoSu group at baseline in continuous normally distributed data, we used unpaired t-tests. For the skewed data, Mann-Whitney U-tests were applied. For categorical data with values below five in any of the cells, Fisher’s exact tests were performed.

Analyses on the inflammatory parameters were performed on a per-protocol approach. Due to the fact that the IL-6 and CRP values were skewed, we made a log-transformation before starting with the analyses over time. Changes within groups were analyzed with paired t-tests; differences between groups were assessed with analysis of covariance (ANCOVA) for repeated measurements. To obtain unbiased results, these analyses were adjusted for baseline value [57]. In addition, we adjusted for sex, age, anti-inflammatory medication, and chronic rheumatism, as these parameters are known to influence inflammatory parameters [58, 59]. Linear regression analyses were applied to examine the association between the baseline values of frailty status, BMI, FMI, ASMMI, phase angle, relative handgrip strength, physical performance, and inflammatory parameters. In these analyses, patients who were lost to follow-up were included, leading to a sample size of 73 persons. As in the analyses over time, these calculations were also adjusted for sex, age, anti-inflammatory medication, and chronic rheumatism. Finally, we performed binary logistic regression analyses to examine whether the baseline values of frailty status, handgrip strength, and physical performance were associated with detected changes in inflammatory parameters. For this purpose, changes in inflammatory parameters were dichotomized into “constant or decreased inflammatory parameters” (≤0.00 units) or “increased inflammatory parameters” (>0.00 units). The dichotomized variable was used as the dependent variable. In the same way, we analyzed, if “constant or decreased inflammatory parameters” were related to “constant or decreased” frailty status (≤0.00 DFS score), “increased” handgrip strength (>0.00 kg), and “increased” physical performance (>0.00 points). These analyses were adjusted for sex, age, anti-inflammatory medication, chronic rheumatism, and group [58, 59]. The analyses were performed with IBM® SPSS® Statistics Version 20 software (IBM Corp., Armonk, NY, U.S.). A p-value of <0.05 was considered to be statistically significant.

## Results

**Fig 1:**
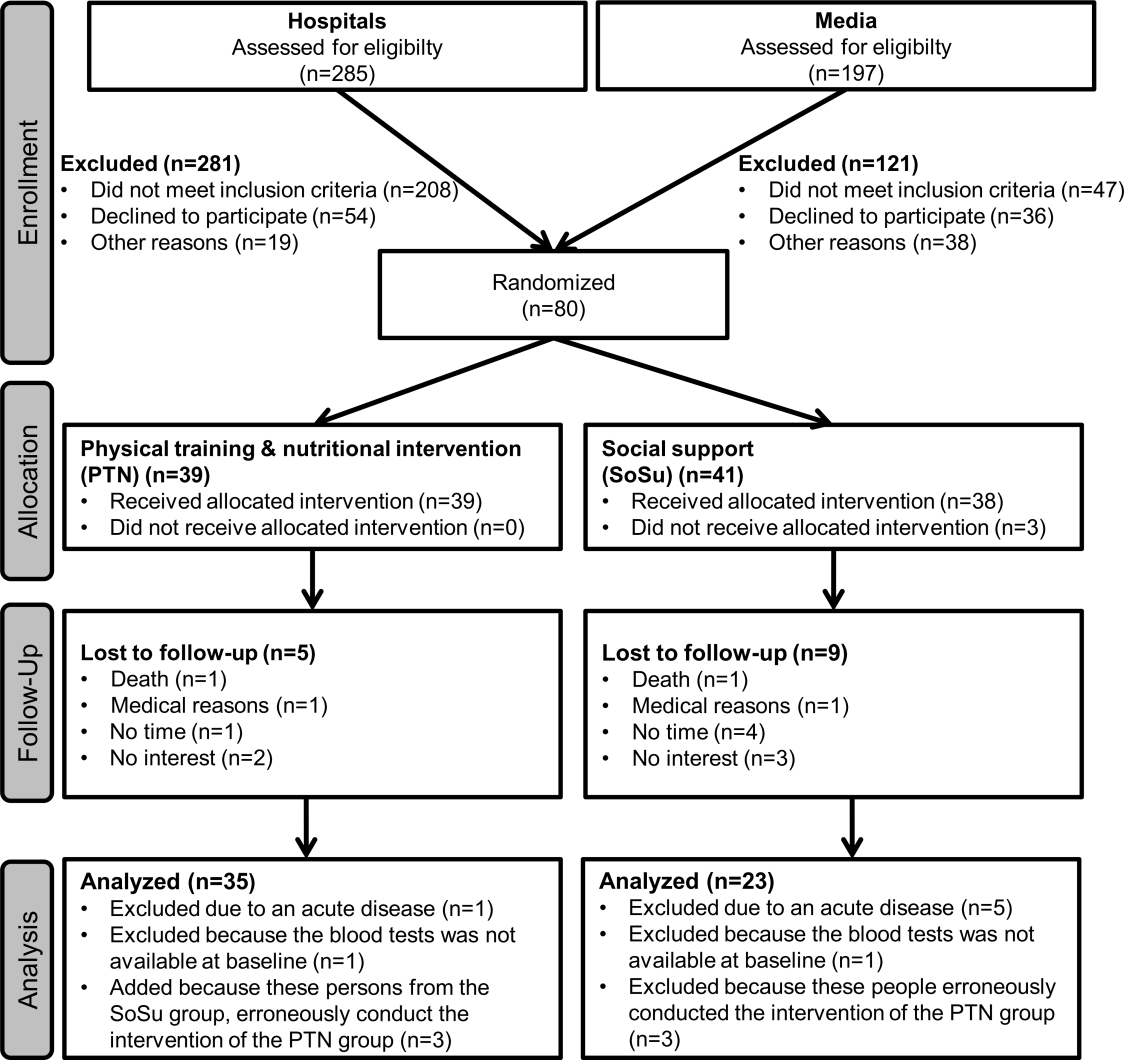
Participant flow.

Fig 1 presents the participant flow.

In total, 482 individuals were checked for eligibility. Only four (5.0%) persons recruited from hospital were included, because 73.0% did not meet the inclusion criteria, 19.2% declined participation, and 6.8% were excluded for other reasons. The remaining 76 (95.0%) adults were recruited with the help of the media. Finally, 39 participants were randomized to the PTN group and 41 to the SoSu group. At the 12-week follow-up, a drop-out rate of 14 (17.5%) subjects was observed, and two (2.5%) participants had to be excluded because the blood tests were not available at baseline. We also excluded six (7.5%) participants due to acute diseases at baseline. Reported diseases were cases of herpes zoster, gastric ulcer, bronchitis, and respiratory infection. In addition, two other (1.6%) participants were excluded because the laboratory tests at baseline were not carried out, due to organizational issues. In addition, two (1.6%) individuals performed physical training and talked about nutrition, although they were assigned to the SoSu group; therefore, they were analyzed in the PTN group. Finally, the 35 members of the PTN group and the 23 members of the SoSu group were analyzed.

The baseline characteristics of the included participants are presented in Table 1.

**Table 1:** Baseline characteristics of the study sample.

	Physical training and nutritional intervention group	Social support group	p-value^a^
	n = 35	n = 23	
Female, n (%)	30 (85.7)	19 (82.6)	0.876
Age (years)	83.1 (7.8)	81.4 (8.7)	0.433
Frailty status (SHARE-FI score)	2.8 (1.1)	2.9 (0.9)	0.664
Nutritional status (MNA®-SF score)	11.2 (2.5)	10.5 (2.5)	0.281
Body mass index (kg/m^2^)	27.5 (5.0)	27.0 (3.0)	0.608
Fat mass index (kg/m^2^)	10.5 (3.5)	10.6 (2.4)	0.936
Appendicular skeletal muscle mass index (kg/m^2^)	6.8 (1.2)	6.3 (0.8)	0.081
Phase angle (°)	4.0 (0.6)	4.0 (0.7)	0.957
Handgrip strength (kg/m^2^)	17.6 (7.4)	16.8 (6.80)	0.687
Physical performance (SPPB score)	5.2 (2.7)	4.3 (3.0)	0.229
**Inflammatory parameters**			
Tumor necrosis factor alpha (pg/ml)	10.2 (3.4)	10.1 (2.7)	0.825
Interleukin-6 (pg/ml)	2.6 (2.0–17.2)	2.6 (2.0–30.6)	0.951
C-reactive protein (mg/dl)	0.2 (0.1–1.7)	0.2 (0.1–5.6)	0.639
Leukocytes total (g/l)	7.0 (2.0)	6.9 (1.7)	0.904
**Comorbidities**			
Arthritis, n (%)	8 (22.9)	2 (8.7)	0.287
Chronic rheumatism, n (%)	4 (11.4)	2 (8.7)	1.000
Cancer, n (%)	3 (8.6)	3 (13.0)	1.000
Hypertension, n (%)	28 (80.0)	15 (65.2)	0.796
Myocardial infarction, n (%)	3 (8.6)	1 (4.3)	1.000
Heart insufficiency, n (%)	9 (25.7)	5 (21.7)	1.000
Stroke, n (%)	6 (17.1)	1 (4.3)	0.226
Diabetes mellitus type 2, n (%)	4 (11.4)	5 (21.7)	0.460
Charlson comorbidity (index)	5.2 (1.5)	5.3 (1.5)	0.618
**Medications**			
Numbers of different drugs per day	8 (0–16)	8 (0–19)	0782
Anti-inflammatory medication, n (%) ^b^	11 (31.4)	5 (21.7)	0.419

Continuous data are presented as mean (standard deviation) or median (minimum–maximum), categorical variables as frequencies (percentages). *SHARE-FI =* Frailty Instrument of the Survey of Health, Ageing and Retirement in Europe; *MNA®-SF* = Mini Nutritional Assessment Short-Form; *SPPB* = Short Physical Performance Battery.

^a^ Values are based on t-tests or Mann-Whitney U-tests (continuous variables) and Fisher’s exact test (categorical variables).

^b^ Including medications with the following chemical agents: ciclesonide, dexibuprofen, diclofenac, fluticasone-propionate, ibuprofen, lornoxicam, mefenamic acid, meloxicam, naproxen, and prednisone.

Among the participants, 84.8% were females, with a mean age of 82.4 years and a standard deviation (SD) of 8.2; 38 (65.5%) were frail; 19 (32.8%) were prefrail; and one (1.6%) was robust but at risk of malnutrition. BMI was calculated as 27.3 (SD = 4.3) kg/m^2^, FMI as 10.6 (SD = 3.1) kg and ASMMI as 6.6 (SD = 1.0) kg. Mean handgrip strength was 17.3 (SD = 7.1) kg, and physical performance came to 4.8 (SD = 2.8) points. In total, 100% of the participants had elevated TNF-α, 100% had elevated IL-6 values, and in 62.1%, CRP levels were increased. Five (8.2%) participants had total leukocyte counts out of the normal ranges. According to the CI, 63.2% of the participants had severe comorbidities. The remaining 36.8% had moderate comorbidities. These participants took a mean average of eight (min–max = 0–19) different drugs per day. There was no difference in any baseline characteristic between those who completed the study and those who were lost to follow-up (data not shown).

Documentation forms showed no difference between the two groups in frequency and duration of the home visits. The PTN group performed a mean average of 1.3 strength circuits (SD = 0.5) consisting of 5.5 exercises (SD = 0.8). A mean average of 12.2 repetitions (SD = 3.9) was conducted. In addition, seven participants (17%) performed strength circuits between the home visits. In respect to nutritional intervention, a mean average of 1 (SD = 0.6) intervention was performed.

The analyses over time, from baseline to 12 weeks, showed that TNF-α increased in 62.1% and total leukocyte count in 60.3% of the participants. Furthermore, IL-6 increased in 62.1% of the individuals, whereas in the SoSu group, an increase from a median value of 2.6 pg/l (min–max = 2.0–10.2) to 3.0 pg/l (min–max = 2.0–20.8) was found. CRP rose in 36.2%, whereas in the SoSu group, an increase from a median value of 0.2 mg/dl (min–max = 0.1–0.9) to 0.3 mg/l (min–max = 0.1–3.0) was seen. Further results showed that, in the PTN group, log-IL-6 and log-CRP remained constant, whereas in the SoSu group, these parameters increased significantly (Table 2). Concerning CRP, we also saw a significant difference between groups. TNF-α and total leucocyte count did not change in either of the groups.

**Table 2:** Changes in inflammatory parameters from baseline to 12 weeks.

		Differences over time^a^		Differences between groups^b^
		Mean change (95% CI)	p-value	p-value
TNF-α (pg/ml)	PTN	−0.32 (−1.24–0.38)	0.287	0.469
	SoSu	−0.47 (−1.40–0.46)	0.303	
Log-IL-6 (pg/ml)	PTN	0.11 (−0.11–0.33)	0.304	0.343
	SoSu	0.19 (0.00–0.37)	0.047	
Log-CRP (mg/dl)	PTN	0.08 (−0.16–0.32)	0.501	0.040
	SoSu	0.46 (0.07–0.85)	0.022	
Leukocytes total (g/l)	PTN	0.48 (−0.04–1.01)	0.070	0.839
	SoSu	0.46 (−0.22–1.13)	0.177	

*PTN* = physical training and nutritional intervention group (n = 35); *SoSu* = social support group (n = 23).

^a^ Differences over time were calculated using paired t-tests. Mean change and 95% confidence interval (95% CI) are presented.

^b^ Differences between groups were calculated using analysis of covariance (ANCOVA) for repeated measurements, adjusted for baseline value, sex, age, anti-inflammatory medication, and chronic rheumatism.

As presented in Table 3, higher frailty status at baseline was associated with higher TNF-α. Lower handgrip strength was related to higher log-IL-6 and better physical performance with lower CRP values. Current BMI, FMI, ASMMI, phase angle, and physical performance were not associated with any inflammatory parameter.

**Table 3:** Association between baseline values of inflammatory parameters and baseline values of body composition parameters, handgrip strength, and physical performance.

	TNF-α (pg/ml)		Log-IL-6 (pg/ml)		Log-CRP* (mg/dl)		Leukocytes total (g/l)	
	ß	0070	ß	p-value	ß	p-value	ß	p-value
Frailty status (SHARE-FI)	**0.360**	**0.002**	0.183	0.117	0.021	0.889	−0.056	0.645
Body mass index (kg/m^2^)	0.173	0.207	0.140	0.302	0.228	0.105	0.188	0.171
Fat mass index (kg/m^2^)	0.241	0.117	0.142	0.353	0.234	0.137	0.243	0.115
ASMMI (kg/m^2^)	0.127	0.357	0.084	0.536	0.218	0.123	0.075	0.589
Phase angle (°)	−0.188	0.137	−0.242	0.052	0.169	0.198	0.038	0.766
Handgrip strength (kg/m^2^)	−0.149	0.233	**−0.237**	**0.047**	0.043	0.748	−0.043	0.732
Physical performance (SPPB)	−0.239	0.051	0.085	0.480	**0.335**	**0.008**	0.177	0.151

In these analyses, patients who were lost to follow-up were also included (n = 73). Values are based on univariate linear regression analyses, with inflammatory parameters as the dependent variables, adjusted for sex, age, anti-inflammatory medication, and chronic rheumatism. Standardized ß-values are reported.

*As the CRP values become negative numbers after log-transformation, associations appearing as positive must be interpreted as negative associations.

*ASMMI* = appendicular skeletal muscle mass index; *SPPB =* Short Physical Performance Battery; *SHARE-FI* = Frailty Instrument of the Survey of Health, Ageing and Retirement in Europe.

Neither frailty status, handgrip strength, nor physical performance at baseline were associated with a higher chance of having “constant or decreased” inflammatory parameters (Table 4). Furthermore, persons who were able to increase their physical performance within the study had a greater chance of having “constant or decreased” log-IL-6 values compared to persons who could not increase physical performance.

**Table 4:** Association between constant or decreased (versus increased) inflammatory parameters within 12 weeks and baseline values/changes in frailty score, handgrip strength, and physical performance.

	Constant or decreased							
	TNF-α		Log-IL-6		Log-CRP		Leukocytes total	
	OR (95% CI)	p-value	OR (95% CI)	p-value	OR (95% CI)	p-value	OR (95% CI)	p-value
BL frailty status (SHARE-FI score)	0.79 (0.43–1.46)	0.447	1.18 (0.66–2.09)	0.570	0.62 (0.30–1.27)	0.194	1.14 (0.65–2.01)	0.642
BL handgrip strength (kg/m^2^)	0.94 (0.83–1.05)	0.265	0.99 (0.89–1.09)	0.801	1.00 (0.88–1.14)	0.978	0.97 (0.88–1.08)	0.591
BL physical performance (SPPB score)	1.09 (0.85–1.39)	0.495	0.96 (0.77–1.20)	0.720	1.16 (0.90–1.51)	0.262	0.97 (0.78–1.21)	0.798
**Δ frailty status (SHARE-FI score)**								
Constant or decreased	1.75 (0.45–6.79)	0.418	1.30 (0.40–4.29)	0.663	2.47 (0.56–10.93)	0.233	0.95 (0.29–3.11)	0.929
Increased	1		1		1		1	
**Δ handgrip strength (kg/m**^2^**)**								
Constant or decreased	1		1		1		1	
Increased	0.52 (0.15–1.84)	0.307	1.29 (0.42–3.93)	0.658	0.66 (0.17–2.51)	0.541	0.63 (0.21–1.93)	0.421
**Δ physical performance (SPPB score)**								
Constant or decreased	1		1		1		1	
Increased	0.56 (0.16–2.03)	0.378	**4.54 (1.33**–**15.45)**	**0.016**	0.49 (0.13–1.87)	0.294	**1.37 (0.45–4.22)**	**0.582**

Values are based on univariate logistic regression analyses, with constant or decreased inflammatory parameters (≤0.00 units) as dependent variables, adjusted for sex, age, anti-inflammatory medication, chronic rheumatism, and group. Odds ratio (OR) and 95% confidence interval (95% CI) are presented. BL = baseline; SHARE-FI = Frailty Instrument of the Survey of Health, Ageing and Retirement in Europe; SPPB = Short Physical Performance Battery, Δ = changes from baseline to 12 weeks.

## Discussion

The findings of this study showed that IL-6 and CRP remained constant in prefrail and frail participants receiving physical training and nutritional support from trained lay volunteers, whereas in those receiving home visits with social support alone, these parameters increased significantly. In addition, further publications of this intervention demonstrated that home visits with physical training and nutritional support can significantly increase handgrip strength and physical performance, but not muscle mass [60]. There was no significant change in these parameters in participants receiving home visits with social support alone.

As very old individuals with severe comorbidities were included in the study, a considerable increase in inflammatory parameters was measured, and two participants died (due to natural death) in the short period of 12 weeks. We are only aware of one comparable study of frail individuals [26]. In this study, obesity was an inclusion criterion, and physical training (three times a week, flexibility, aerobic, resistance, balance) in addition to an energy-deficit diet was conducted. Although FM decreased, no change in IL-6 and TNF-α was detected. With respect to this study, and based on the fact that in our study FM remained stable with an increase in IL-6 and CRP in the SoSu group, it can be concluded that a change in FM might not be the cause for the change in inflammatory parameters in prefrail and frail persons. The finding that physical and nutritional training can influence CRP and IL-6, with no effect on TNF-α, is in line with the study of Nicklas et al. [61] conducted in older overweight and obese individuals. In addition, the study participants of Nicklas et al. [61] conducted an exercise program consisting of aerobic and strength training, while our study participants performed strength training only. These different training methods might also affect inflammatory parameters in a different way. Despite this fact, the main difference between their intervention and our study is that the intervention of Nicklas et al. [61] led to a reduction in body weight, whereas in our study, body weight remained stable. This is in addition to the fact that they saw a decrease in CRP and IL-6, whereas we only saw stable values in the PTN group. As, in our study, the CRP and IL-6 of the SoSu group increased with significant between-group differences, we are confident that, in very old individuals with advanced frailty status, stable inflammation values may be considered a success. In line with the justification mentioned in Nicklas et al. [61], the non-significant change in TNF-α might be traced back to the transient production and the short half-life [62].

Reasons for the significant differences between the PTN group and the SoSu group can only be vaguely formulated. The first possible reason, which has been postulated in earlier research, although considered as rather unlikely, is that a reduction in adipose mass or BMI might lead to a decrease in IL-6 and consequently to decreased CRP production [24]. This hypothesis is not supported by our data, as we did not see any significant change in BMI and FM. The second possible reason, as suggested by Geffken et al. [23], is that physical activity might reduce inflammation by improving insulin resistance. Unfortunately, in the present study, insulin resistance was not assessed. The third possible reason might be that physical activity influences endothelial cells, which secrete IL-6 [63]. With the data of the present study, no statement can be made about this hypothesis. The same applies to a fourth possible reason, which is the suggestion that the downregulation of toll-like receptor-4 in the skeletal muscle might lead to a decrease in mRNA, and declining TNF-α and IL-6 [26]. A fifth possible reason could be that the PTN group increased the uptake of anti-inflammatory nutrients such as polyphenols. However, this hypothesis is rather unlikely as no increase in the fruit and vegetable intake or the Mediterranean diet score [64] was found (data not yet published).

The non-association between physical performance, muscle mass, and inflammatory parameters is not surprising. These results are in line with the findings of Legrand et al. [13] and Cesari et al. [14]. In addition, in older high-functioning persons, the MacArthur study [15] showed only limited association between physical performance and inflammatory parameters. The authors claimed that the diversity in functional limitations was responsible. Although we included a more homogeneous group, we still did not see a significant association, leading to the conclusion that diversity in disabilities might not be the main reason for the non-association. Our results also showed that in prefrail and frail persons, phase angle is not associated with inflammatory parameters, although it has been shown to be an indicator of cellular death in elderly individuals [65]. Thus, it might be postulated that in elderly persons with poor general health, phase angle is not related to inflammatory parameters. Oxidative stress has been reported to have an association with frailty [66]. However, from the results of our study, no statement about the association between oxidative stress (e.g. lipoprotein phospholipase, isoprostanes) or anti-oxidant parameters (e.g. vitamins C, E, tocopherol, total thiol levels) and frailty can be made.

To the best of our knowledge, this is the first study of its kind to investigate the effects of physical training and nutritional support on inflammatory parameters in a sample of prefrail and frail community-dwelling older persons, which we consider to be a major strength. In addition, it is the first time that such an intervention was provided by lay volunteers. Despite these strengths, the study also has some limitations. As the sample size calculation is based on the main outcome of handgrip strength, the effects on inflammatory parameters might be underpowered. Furthermore, the small number of participants and the relatively high number lost to follow-up is a further limitation. ASMM and FM were assessed with a BIA device. However, as a distorted hydration status might influence the results, this can be considered as a disadvantage. The fact that IL-6 and CRP have to be log-transformed also made the data difficult to interpret. Although we tried to adjust for possible confounders, it cannot be ruled out that other confounders influence inflammatory parameters. Moreover, it would be better to use highly sensitive CRP. Another limitation is that insulin concentration and mRNA were not assessed. These parameters could give more information about the achieved group differences.

## Conclusions

Prefrail and frail older adults should be encouraged to take part in a standardized strength training program in combination with nutritional support provided by lay volunteers, as it might prevent or delay a further increase in IL-6 and CRP. These results are clinically important, as inflammatory parameters are associated with a further decline in skeletal muscle mass, and these parameters predict mortality. An increase in physical performance is particularly important, as it is associated with a higher chance of reaching constant or decreased log-CRP values.

Further research is needed to examine the physiological process behind the effects of physical training and nutritional support on inflammatory parameters in prefrail and frail older adults.

## Supporting information

S1 Dataset Dataset from the Medical University of Vienna. (SAV)

S1 File CONSORT checklist. (DOC)

S2 File Ethic vote. (PDF)

S3 File Impact of a home-based physical and nutritional intervention program conducted by lay-volunteers on handgrip strength in prefrail and frail older adults: A randomized control trial. (PDF)

S4 File Study protocol. (PDF)
