# Supplementary material for: Change in inflammatory parameters in prefrail and frail persons obtaining physical training and nutritional support provided by lay volunteers: A randomized controlled trial
Source: PLoS One. 2017 Oct 12;12(10):e0185879. doi: 10.1371/journal.pone.0185879 (PMC5638289; doi:10.1371/journal.pone.0185879)
Supplement: S2 File — (PDF) [file pone.0185879.s003.pdf]

ethik kommission
Medizinische Universität Wien

Borschkegasse 8b/6
A-1090 Vienna, Austria
T +43 1 40 400 – 2147, 2244
F +43 1 40 400 – 1690
ethik-kom@meduniwien.ac.at
ethikkommission.meduniwien.ac.at

Vote:

EK Nr: 1416/2013
Project title: Nutrition intervention and physical training in malnourished frail community-dwelling elderly subjects carried out by trained lay "buddies"
Applicant: Sandra Haider
Institution: Medical University of Vienna/ Centre for Public Health
Sponsor: Medical University of Vienna/ Centre for Public Health

Involved centres:

**Ethics committee**	**Trial center**	**Investigator**
Ethics committee of Medical University of Vienna	Department of Internal Medicine III, Division of Endocrinology and Metabolism	Ao.Univ.-Prof. Dr.med.univ. Bernhard Ludvik

The statement of the ethics committee is due to the following submitted documents:

**Document**	**Name**	**Version**	**Date**
Case report form (CRF)	CRF_Buddies_1.0_24042013	1.0	24.04.2013
	CRF_Frailty_1.0_24042013	1.0	24.04.2013
Conflict of Interest	ConflictOfInterest_22042013	1.0	22.04.2013
CV	CV_BernhardLudvik_26.04.2013	1.0	26.04.2013
	Lebenslauf_Dorner_24042013		24.04.2013
	Lebenslauf_Schindler_24042013		24.04.2013
	Lebenslauf_Lackinger_24042013		24.04.2013
	Lebenslauf_Luger_24042013		24.04.2013
	Lebenslauf_Kapan_24042013		24.04.2013
	Lebenslauf_Prenner_24042013		
	Lebenslauf_Oberbauer_24042013		
	Lebenslauf_Haider_24042013		
Confirmation of cover	Versicherungsbestätigung_1.1_22082013	1.1	22.08.2013
	Versicherungsbestätigung_1.0	1.0	02.04.2013
	Versicherungsbestätigung-Buddies-Haftpflicht_1.0	1.0	16.08.2011
	Versicherungsbestätigung-Buddies_Unfall_1.0	1.0	29.06.2011
Other	Verpflichtungserklaerung_Haider	1.0	25.04.2013
	Verpflichtungserklaerung_Kapan	1.0	25.04.2013
	Verpflichtungserklaerung_Luger	1.0	25.04.2013
	Verpflichtungserklaerung_Prenner	1.0	25.04.2013
	Fragebogen_1.0_24042013	1.0	24.04.2013
Patient information	PatientInneninformation_Buddies_1.3_markiert_21082013	1.3	21.08.2013

	PatientInneninformation_Buddies_1.3_21082013	1.3	21.08.2013
	PatientInneninformation_Buddies_1.2_markiert_25072013	1.2	25.07.2013
	PatientInneninformation_Buddies_1.2_25072013	1.2	25.07.2013
	PatientInneninformation_Frailty_1.2_24072013	1.2	24.07.2013
	PatientInneninformation_Frailty_1.2_markiert_24072013	1.2	24.07.2013
	PatientInneninformation_Frailty-Rekrutierung_1.1_markiert_08072013	1.1	08.07.2013
	PatientInneninformation_Frailty-Rekrutierung_1.1_08072013	1.1	08.07.2013
	PatientInneninformation_Frailty_1.1_markiert_08072013	1.1	08.07.2013
	PatientInneninformation_Frailty_1.1_08072013	1.1	08.07.2013
	PatientInneninformation_Buddies_1.1_08072013	1.1	08.07.2013
	PatientInneninformation_Buddies_1.1_markiert_08072013	1.1	08.07.2013
	PatientInneninformation_Buddies_1.0_24042013	1.0	24.04.2013
	PatientInneninformation_Frailty_1.0_24042013	1.0	24.04.2013
	PatientInneninformation_Frailty-Rekrut_1.0_24042013	1.0	24.04.2013
Study protocol	StudyProtocol_1.1_markiert_08072013	1.1	08.07.2013
	StudyProtocol_1.1_08072013	1.1	08.07.2013
	Studienprotokoll_1.0_24042013	1.0	24.04.2013

The commission adopts the following resolution (with X marks):

☒	There is no objection to conduct the study.

**Signaturwert**	biE5HK0UAEwTln7deNLKHhYIhzDAGk+SczDoSCAejdyAD7EdXJ66Qq0oak+v3wgx1VQs46Yz8nWwemJFvn8yuQ==	
	**Unterzeichner**	Dr. Ernst Singer
	**Aussteller-Zertifikat**	CN=a-sign-Premium-Sig-02,OU=a-sign-Premium-Sig-02,O=A-Trust Ges. f. Sicherheitssysteme im elektr. Datenverkehr GmbH,C=AT
	**Serien-Nr.**	456499
	**Methode**	urn:pdfsigfilter:bka.gv.at:binaer:v1.1.0
	**Parameter**	etsi-moc-1.1@7806fc14
**Prüfinformation**	Informationen zur Prüfung der elektronischen Signatur und des Ausdrucks finden Sie unter: http://www.signaturpruefung.gv.at	
**Datum/Zeit-UTC**	2013-08-27T13:44:27Z	

Signature of the applicant

Mag. **Sandra Haider**
Medical University of Vienna/ Centre for Public Health
Kinderspitalgasse 15/1
1090 Vienna, Austria
