# Supplementary material for: Change in inflammatory parameters in prefrail and frail persons obtaining physical training and nutritional support provided by lay volunteers: A randomized controlled trial
Source: PLoS One. 2017 Oct 12;12(10):e0185879. doi: 10.1371/journal.pone.0185879 (PMC5638289; doi:10.1371/journal.pone.0185879)
Supplement: S3 File — (PDF) [file pone.0185879.s004.pdf]

PLOS | ONE

RESEARCH ARTICLE

Impact of a Home-Based Physical and Nutritional Intervention Program Conducted by Lay-Volunteers on Handgrip Strength in Prefrail and Frail Older Adults: A Randomized Control Trial

Sandra Haider[1]*, Thomas E. Dorner[1], Eva Luger[1,2], Ali Kapan[1], Sylvia Titze[3], Christian Lackinger[4], Karin E. Schindler[5]

1 Institute of Social Medicine, Centre for Public Health, Medical University of Vienna, Vienna, Austria, **2** Special Institute for Preventive Cardiology And Nutrition (SIPCAN), Salzburg, Austria, **3** Institute of Sport Science, University of Graz, Graz, Austria, **4** SPORTUNION Austria, Department for Health Promotion and Prevention, Vienna, Austria, **5** Department of Internal Medicine III, Division of Endocrinology and Metabolism, Medical University of Vienna, Vienna, Austria

* sandra.a.haider@meduniwien.ac.at

Check for updates

OPEN ACCESS

Citation: Haider S, Dorner TE, Luger E, Kapan A, Titze S, Lackinger C, et al. (2017) Impact of a Home-Based Physical and Nutritional Intervention Program Conducted by Lay-Volunteers on Handgrip Strength in Prefrail and Frail Older Adults: A Randomized Control Trial. PLoS ONE 12(1): e0169613. doi:10.1371/journal.pone.0169613

Editor: Yoshihiro Fukumoto, Kurume University School of Medicine, JAPAN

Received: September 28, 2016

Accepted: December 15, 2016

Published: January 13, 2017

Copyright: © 2017 Haider et al. This is an open access article distributed under the terms of the Creative Commons Attribution License, which permits unrestricted use, distribution, and reproduction in any medium, provided the original author and source are credited.

Data Availability Statement: All relevant data are within the paper and its Supporting Information files.

Funding: This work was supported by the 'Vienna Science and Technology Fund' (grant number: LS12-039). The sponsors played no role in the study design, the methods, participant recruitment, data collection, analysis, or preparation of the paper.

Abstract

A randomized controlled trial was performed to compare the effects of a home-based physical and nutritional intervention program carried out by lay-volunteers to home visits with social support alone. Buddies visited 80 prefrail or frail older persons at home twice a week for 12 weeks. The physical training and nutrition group (PTN, n = 39) performed two sets of six strength exercises, discussed nutritional topics and received social support. The social support group (SoSu, n = 41) received home visits with social support only. In the PTN group, handgrip strength increased significantly by 2.4 kg (95% CI: 1.0–3.8). In the SoSu group we did not see a significant improvement. However, no significant between-group difference was found. Physical performance increased in both groups, although with a higher increase of 1.0 point (95% CI: 0.1–2.0) in the PTN group. In none of the groups muscle mass changed. Further results showed that frail individuals benefit more from the intervention than prefrail individuals (OR: 2.78; 95% CI: 1.01–7.66). Handgrip strength in the intervention group increased by a clinically relevant value and this effect is comparable to that obtained by health-care professionals. Therefore, home visits with a physical training and nutritional program could offer a new perspective in the care of community-dwelling prefrail and frail older persons.

Introduction

Frailty, a geriatric syndrome, is characterized by a decrease in biological functions [1] and a high susceptibility for adverse health outcomes [2]. As sarcopenia and malnutrition contribute to the frailty syndrome [3], strength training, in combination with a nutritional intervention program, is an effective way to tackle frailty [4–6]. It has therefore been recommended that

Competing Interests: The authors have declared that no competing interests exist.

prefrail and frail adults perform 6–10 strength exercises with 1–3 sets and 18–20 repetitions in circuit form at least twice a week [7]. As nutritional status is a mediating factor of frailty [8], and a balanced diet is an important requirement to minimize the age-related decline of muscle strength and muscle mass, a sufficient energy, protein, and micronutrient intake is required [9, 10].

In prefrail and frail persons, the effects of strength training supervised by health-care professionals have already been extensively studied. In these trials muscle strength was mainly measured by handgrip strength or by strength in the knee extensor. Depending on the protocol (e.g. frequency, duration, number of exercises, sets, intensity, repetitions), some of the interventions were successful in improving muscle strength, whereas others were not [11–15]. Current evidence suggests that also peer-delivered training interventions can help to increase physical activity behavior in older subjects [16, 17]. In the systematic review of Ginis et al. [18], it has even been postulated that these interventions are as effective as interventions conducted by health-care professionals.

In several publications the impact of nutritional supplementation has been investigated. However, the majority of these studies failed to increase muscle strength [4, 13, 19, 20]. Furthermore, findings from previously published studies showed that nutritional supplementation in combination with strength training did not increase muscle strength more than strength training alone [13, 14, 21, 22]. The effect of nutritional consultation on muscle strength has rarely been studied. In malnourished individuals, however, it was found that nutritional consultation alone did not improve handgrip strength [23]. To your knowledge, studies examining the combination of nutritional consultation and physical training are broadly missing.

As in the upcoming years the number of prefrail and frail individuals will further increase and frailty will become a huge public health issue [24], we were interested in the effects of home visits provided by volunteers. To that end, we compared the effects of a home-based physical and nutritional intervention program provided by trained lay-volunteers to home visits with social support alone on our primary outcome variable handgrip strength. Additionally, physical performance and muscle mass were investigated as secondary outcome variables.

Materials and Methods

Study design

The study protocol of this randomized controlled trial, which was carried out from September 2013 to July 2015 in Vienna, Austria, has already been published [25]. Based on a sample size calculation (two-sided t-test, significance level: 0.05, power: 80%, drop-out: 20%) with a clinically relevant difference in handgrip strength between the two study groups of ≥2 kg and a standard deviation (SD) of 3 kg, in total 80 prefrail and frail older adults were included. The randomization, which was stratified by handgrip strength, was done with the randomizer called "Randomizer for Clinical Trials 1.8.1" [26]. For this purpose, sex-specific cut-off values (male: 22 kg; female: 15 kg), which were based on the median value of our pre-study [27], were used.

The research complied with the Declaration of Helsinki [28]. Ethical clearance was given by the local ethical committee of the Medical University of Vienna (Ref: 1416/2013), and written informed consent were obtained from all included study participants. The protocol was registered at clinicaltrials.gov (identifier: NCT01991639).

Study participants

Prefrail and frail individuals. Individuals fulfilling the following inclusion criteria were recruited [25]: Subjects had to be older than 65 years and they had to live in their own homes.

In addition, subjects had to be at least prefrail according to the "Frailty Instrument of the Survey of Health, Ageing and Retirement in Europe" (SHARE-FI) [29] or they had to be at least at risk of malnutrition according to the "Mini Nutritional Assessment Short-Form" (MNA®-SF ≤11 points) [30]. The SHARE-FI is an age- and sex-specific calculator including five items (exhaustion, loss of appetite, weakness, slowness, and low physical activity). Based on a discrete factor score (DFS), this calculator divides persons into robust, prefrail, and frail. In addition, individuals with impaired cognitive function according to the "Mini Mental State Examination" (MMSE ≤17 points) [31], insufficient German language skills, chemo- or radio-therapy at the moment or planned, insulin-treated diabetes mellitus, chronic obstructive pulmonary disease stage III or IV, or chronic kidney insufficiencies with protein restriction were excluded. Persons with a medical contraindication for performing strength training were also excluded.

At the beginning of the study, the recruitment was done in three hospitals, in wards for internal medicine. Over the course of the study, interested persons responded to one editorial feature in the local newspaper "Kurier" and to one television report, presenting the content of the study.

Lay volunteers. The following inclusion criteria were applied for the lay volunteers, called 'buddies': Persons had to be older than 50 years and willing to conduct two home visits weekly. These volunteers were recruited in cooperation with the "Wiener Hilfswerk", a well-known social organization in Austria, having experience with volunteers. Before the buddies were included in the study they got detailed information about the study and the associated obligations in a one-hour meeting. Additionally, the buddies underwent a structured interview with a psychologist, checking for motivation and their intention for participation. Moreover, interested persons had to bring a certificate of good conduct.

Intervention

At the beginning, the included buddies were trained four times for approximately three hours each session. In these lessons, the issues of aging, frailty, and malnutrition were discussed and strategies how to motivate older people were presented. Moreover, the buddies were trained in the implementation of the physical and nutritional intervention program. In addition, the documentation form, in which the details of each home visit had to be recorded, was explained. A detailed description of these training sessions has been previously published [25]. After these training sessions, each buddy was allocated to one prefrail or frail individual, dependent on the place of residence. The study groups differed in the following ways:

- Physical training and nutrition (PTN) group: Individuals belonging to the PTN group performed physical training in addition to a nutritional intervention program. Accordingly, each home visit comprised a warm-up with mobilization exercises followed by six standardized strength exercises performed in circuit form [25]. The included strength exercises were: mini squats in front of a chair; chest presses against elastic resistance; an exercise for the abdominal muscles, performed sitting on a chair; hip extensions in standing position; and reverse butterfly and shoulder presses against elastic resistance. To conduct these exercises, study participants were issued with an elastic resistance band and a guidebook describing the exercises. These exercises were performed in two sets of 15 repetitions until muscular exhaustion. If an exercise was not possible (e.g. due to physical limitations), this exercise was skipped. If less than 15 repetitions were possible at the beginning, first of all the numbers of repetitions was increased. Afterwards, exercise intensity was progressively increased by adapting the resistance of the elastic band. Buddies were also asked to encourage the prefrail and frail individuals to conduct the strength exercises once a week alone. As described in the study protocol [25], it was planned that the buddies train simultaneously with prefrail and

frail subjects.
Additionally, a nutritional intervention program was conducted. This intervention consisted of eight nutritional issues, focusing mainly on fluid, protein, and energy intake. During each home visit, one nutritional issue was discussed. As an assistive device for the implementation, participants were provided with a handbook covering all eight nutritional themes. In addition, participants were provided with the "Healthy for Life Plate", which is a modification of the "Health Eating Plate of the Harvard University" [32], and they were also issued with a recipe book, which includes protein-rich dishes [25].
Within these home visits prefrail and frail individuals also received social support.

- Social support (SoSu) group: Individuals in the SoSu group only received social support, without conducting the physical training and nutritional intervention program. Instead, participants of this group were engaged in conversation or performed cognitive training with the help of a guidebook.

During the whole trial, buddies, irrespective of their group assignment, were able to call health-care professionals (sports and nutrition scientists, physiotherapist, dietician or psychologist). Additionally, to exchange experiences, to discuss open issues and, to seek for support buddies could also attend one so-called 'buddy meeting'.

Measurements

In order to assess the effects of the intervention, the study team performed the following measurements at the participants' home environment at baseline and after 12 weeks [25].

- *Personal data*, including sex, age, and living arrangement (living alone, living with others), and *individuals' medications* were recorded at baseline. Additionally, *comorbidities* were assessed by self-reports and the "Charlson comorbidity index" was calculated by summing the weighted comorbidities [33].
- *Handgrip strength* was assessed using a hydraulic hand dynamometer from Jamar® (Lafayette, Louisiana), with the individual in a sitting position [34]. The strength of each hand was alternately tested two times, with a break of 1 minute in between. The highest of all four values in kg was taken for the calculation. Handgrip strength was also categorized based on the mean values of frail individuals reached in the SHARE-study (female: $\geq$17.9 kg and $<$17.9 kg; male: $\geq$26.5 kg and $<$26.5 kg) [29].
- *Physical performance* was measured with the "Short Physical Performance Battery" (SPPB) [35]. The SPPB assesses physical performance in three sub-categories: balance, gait speed, and lower limb muscle strength. Accordingly, balance is measured with a side-by-side, semi-tandem, and tandem stand, gait speed with a 4-m walk (static start, no deceleration at the end), and lower limb muscle strength with the time taken to stand up and return five times. Finally, a performance score, summing all three categories, was calculated. The range of possible scores was from 0 (lowest) to 12 (highest).
- *Lean body mass* and *appendicular skeletal muscle mass* were assessed with a bioelectrical impedance analysis "BIA 2000-S device" from Data Input® (Darmstadt, Germany). Individuals had to lie on their back, with hands and feet 45° apart. Four electrodes were then stuck on the dominant hand and the dominant foot. With the help of an alternating current, body resistance and reactance were assessed [36]. Lean body mass (kg) was calculated with the formula 'total body water/0.73' [37]. Appendicular skeletal muscle mass (kg) was assessed with the validated formula of Sergi and colleagues. [38]. Body height was measured with a tape,

and body weight with the Marsden MS-4203® (Rotherham, UK) calibrated scale. Additionally, relative values of lean body mass and appendicular skeletal muscle mass (kg/m^2) were calculated by dividing the results by the body height squared.

- The *drop-out rate* was analyzed discriminating between 'lost to follow-up' (death and medical reason) and 'discontinued intervention' (no time, no interest).
- Every *adverse event* was documented by the study team. The following standardized procedure was used: Whenever the health status of prefrail or frail individual changed massively, making the intervention impossible for more than one week, buddies called the study team. They gather more information (e.g. medical findings). If the continuation of the intervention was not possible anymore, the study team divided the adverse events into 'caused by the intervention' and 'not caused by the intervention'.
- *Adherence* was evaluated with the following parameters, which were recorded by the buddies on the documentation forms: frequency and duration of each home visits, number of sets per home visit, number of conducted strength exercises per home visit, number of repetitions, and number of circuits completed between the home visits. In these analyses, only persons who had not dropped out were included.

Statistical methods

Differences at baseline between the PTN and SoSu groups in continuous variables were tested with unpaired t-tests or Mann-Whitney U-tests. Chi-square tests or Fisher's exact tests were used to test the differences in the categorical variables.

The effects of the intervention on the outcome values were analyzed according to the intention-to-treat principle, meaning that all randomized patients were included irrespective of whether the intervention was completed or not. In cases where data were not available after 12 weeks (e.g. lost to follow-up, discontinued intervention), the last observation carried forward (LOCF) method was used, imputing the last observed values [39]. Before doing the analyses over time, the distribution of the data was checked visually with histograms and box plots. As the data were normally distributed, within-group differences (from baseline to 12 weeks) were assessed with paired t-tests. Between-group differences (PTN compared to SoSu group) were determined using analysis of covariance (ANCOVA) for repeated measurements, adjusted for sex, age, and baseline values. Sex and age were chosen as control variables, since they influence handgrip strength [40]. To ensure unbiased results the outcome parameters were also adjusted for the corresponding baseline values (e.g. change in handgrip strength was adjusted for baseline value in handgrip strength) [41]. In addition, the percentage change of the main outcome parameter—handgrip strength—was calculated using the formula (12 weeks–baseline value)/baseline value*100. We also conducted a post-hoc power-analysis of handgrip strength to assess if the calculated sample size of 80 persons was actually adequate to show a difference between the PTN and the SoSu group. In order to evaluate if there were baseline characteristics (e.g. sex, age, living arrangement, frailty and nutritional status, handgrip strength, physical performance, BMI, lean body mass, appendicular muscle mass, and Charlson comorbidity index) associated with an improvement in handgrip strength, we applied univariate logistic regression analyses. For this purpose, we classified the participants in persons showing an improvement in handgrip strength and persons not showing an improvement and showing a decrease in handgrip strength, respectively. Since we had defined an improvement of $\geq$2 kg as clinically relevant in the study protocol [25], we have chosen 2 kg as a cut-off point (dependent variable). In this analysis, handgrip strength was categorized based on the mean values of frail

individuals reached in the SHARE-study, (female: ≥17.9 kg and <17.9 kg; male: ≥26.5 kg and <26.5 kg).[29]. Baseline characteristics were included as the independent variables. For all the statistical analyses, IBM® SPSS® Version 20 software (IBM Corp., Armonk, NY, U.S.) was used. All the tests were two-sided, and a p-value of <0.05 was considered to be statistically significant.

Results

Study participants and work flow

As shown in Fig 1, 285 hospitalized patients were checked for eligibility. Of this total, 73.0% did not fulfill the inclusion criteria, 70.1% of the people fulfilling the inclusion criteria declined participation, and 24.7% were excluded for other reasons. Finally, four patients from hospitals, amounting to 1.5%, participated in the study. The remaining 76 individuals (95.0%) were recruited via the media with two editorial features.

Baseline characteristics of the included participants are shown in Table 1. In total, 67 (83.8%) persons were female and the participants had a mean age of 82.8 (SD: 8.0) years, ranging from 65 to 97 years. According to the SHARE-FI, 52 (65.0%) adults were frail, 27 (33.8%) were prefrail, and one (1.2%) participant was robust. In addition, 38 (47.5%) individuals were at risk of malnutrition or malnourished. Mean handgrip strength was 16.3 kg (SD: 7.0). The included participants obtained a physical performance score of 5.0 points (SD: 2.9) and had an appendicular skeletal muscle mass of 17.0 kg (SD: 3.3). No significant difference was observed between the PTN and SoSu groups.

Changes associated with the intervention

In the PTN group, we found a significant improvement in handgrip strength by 2.4 kg (95% CI: 1.0–3.8) amounting to 21.6% of the baseline value. There was also an increase in physical performance, more precisely in balance skills and in the rate of the five timed chair stands (Table 2). Gait speed, lean body mass and appendicular muscle mass did not change significantly.

In the SoSu group, we saw a significant improvement in physical performance, more precisely in the balance skills, whereas all other variables did not change. When comparing the changes of the PTN group to the changes of the SoSu group, we detected a significant between-group difference in physical performance and the lower limb muscle strength. No between-group difference was seen in the primary outcome handgrip strength. This analysis had a statistical power of beta = 0.928, according to a post-hoc power-analysis. Furthermore, no between-group difference was found in balance score, lean body mass and appendicular skeletal muscle mass.

Further results showed that 35 (43.8%) of all individuals, and 21 (53.8%) participants of the PTN group, were able to improve handgrip strength ≥2 kg. As Table 3 shows, frail individuals had a higher chance of improvement than prefrail persons. Results also show that baseline values of sex, age, living arrangement, nutritional status, physical performance, BMI, lean body mass, appendicular skeletal muscle mass and Charlson comorbidity index were not associated with an improvement in handgrip strength.

Drop-out rate, adverse events, and adherence

The drop-out rate is presented in Fig 1. Accordingly, 14 persons dropped out, amounting to 18% of the baseline population. In total, four adverse events (0.5%) 'not caused by the intervention' occurred: two older adults died and two persons interrupted the study for medical reasons. In addition, 10 individuals (1.3%) discontinued the intervention in the first two weeks.

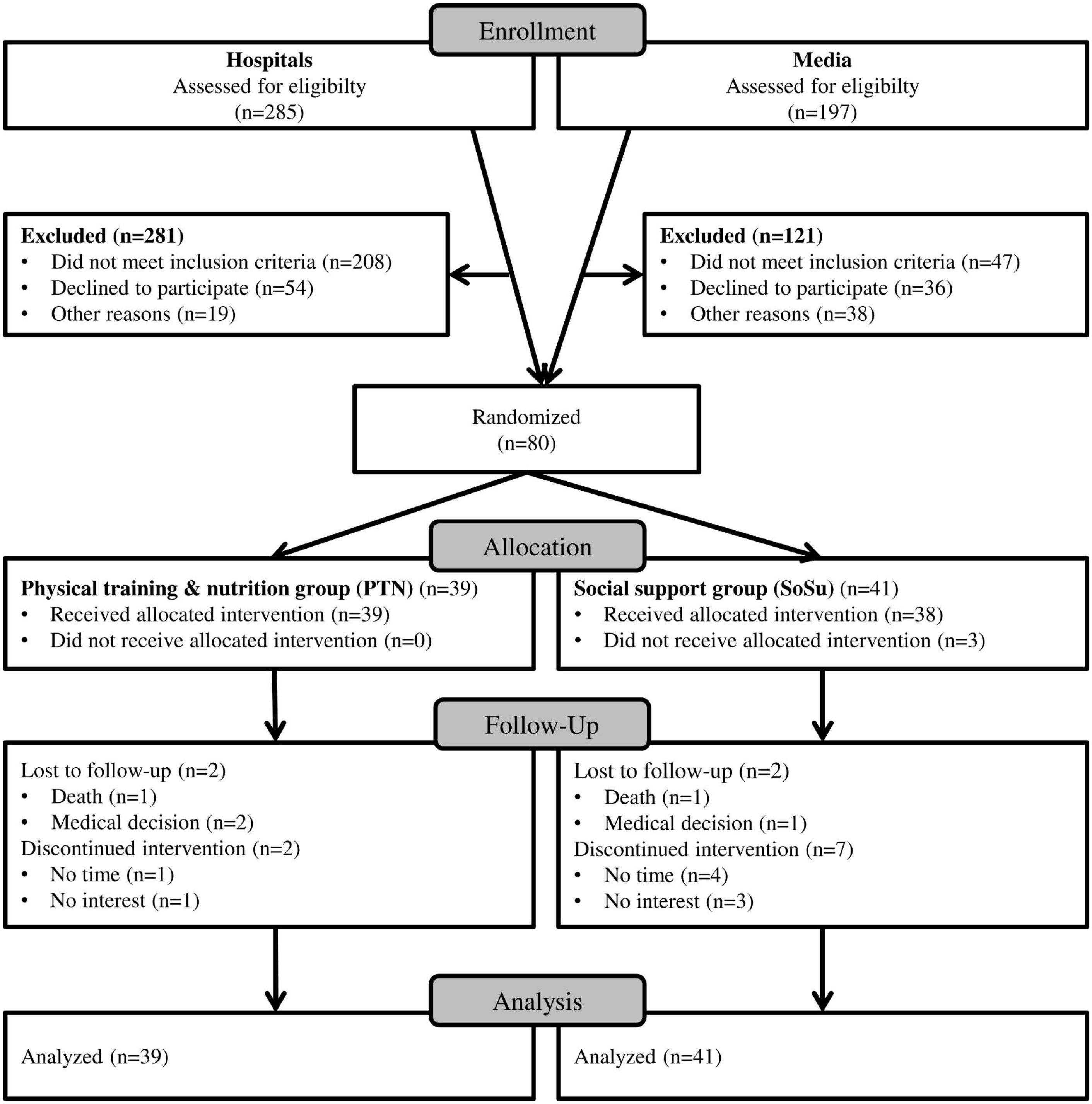

Fig 1. Flow chart of the participants.

doi:10.1371/journal.pone.0169613.g001

Moreover, seven (8.8%) prefrail or frail subjects replaced their buddies for the following reasons: illness of the buddy (n = 3; 3.8%); buddies and frail did not harmonize (n = 4; 5.0%).

Documentation forms describing the content of each home visit were obtained from 65 participants. Frequency and duration of the home visits did not differ between the PTN and

Table 1. Baseline characteristics of study participants in each group.

	Physical training and nutrition group	Social support group	p-value [a]
	n = 39	n = 41	
**Sex**			
Female, n (%)	33 (84.6)	34 (82.9)	0.838
**Age (years)**	83.0 (8.0)	82.5 (8.0)	0.775
**Living arrangement**			
Living alone, n (%)	27 (69.2)	33 (80.5)	0.305
**Frailty status (SHARE-FI score)**	2.9 (1.1)	2.8 (0.9)	0.497
Robust, n (%)	1 (2.6)	0 (0.0)	0.728
Prefrail, n (%)	14 (35.9)	13 (31.7)	
Frail, n (%)	24 (61.5)	28 (68.3)	
**Nutritional status (MNA®-SF score)**	10.9 (2.7)	11.1 (2.5)	0.729
Normal nourished, n (%)	21 (53.8)	21 (51.2)	0.778
Risk of malnutrition, n (%)	14 (35.9)	17 (41.5)	
Malnourished, n (%)	4 (10.3)	3 (7.3)	
**Handgrip strength (kg)**	15.6 (7.0)	17.0 (7.0)	0.960
≥17.9 kg in female, ≥26.5 kg in men, n (%) [b]	13 (33.3)	14 (34.1)	0.939
<17.9 kg in female, <26.5 kg in men, n (%) [b]	26 (66.6)	27 (65.8)	
**Physical performance (SPPB score)**	5.2 (2.9)	4.8 (2.8)	0.559
Balance (score)	2.1 (1.4)	2.1 (1.2)	0.784
Gait speed (score)	1.9 (1.0)	1.9 (1.0)	0.849
Lower limb muscle strength (score)	1.0 (0.0–4.0)	0.0 (0.0–4.0)	0.115
**BMI (kg/m^2)**	27.1 (4.6)	27.6 (4.3)	0.637
**Lean body mass absolute (kg)**	50.53 (8.67)	48.40 (6.92)	0.248
Lean body mass relative (kg/m^2)	19.10 (2.25)	18.32 (1.92)	0.117
**Appendicular muscle mass (kg)**	17.26 (3.62) [c]	16.73 (3.05) [d]	0.498
Appendicular muscle mass relative (kg/m^2)	6.51 (0.98) [c]	6.31 (0.79) [d]	0.346
**Comorbidities**			
Cardiac insufficiency, n (%)	8 (20.5)	10 (24.4)	0.678
Hypertension, n (%)	29 (74.4)	30 (73.2)	1.000
Diabetes mellitus type 2, n (%)	5 (12.8)	8 (19.5)	0.594
Chronic rheumatism, n (%)	5 (12.8)	2 (4.9)	0.258
Morbus Parkinson, n (%)	3 (7.7)	3 (7.3)	1.000
Charlson comorbidity index	5.2 (1.5)	5.3 (1.5)	0.794

Data are presented in mean (standard deviation), median (minimum–maximum), or frequencies (percentages).
MMSE = Mini Mental State Examination, *SHARE-FI* = Frailty Instrument of the Survey of Health, Ageing and Retirement in Europe, *MNA®-SF* = Mini nutritional assessment-Short form, *SPPB* = Short Physical Performance Battery
[a] Continuous data: t-tests or Mann-Whitney U-tests; categorical data: Chi-square tests or Fisher's exact tests
[b] Based on the mean values of frail individuals in the SHARE-study [29].
[c] n = 38, due to missing bioelectrical impedance analyses
[d] n = 35, due to missing bioelectrical impedance analyses

doi:10.1371/journal.pone.0169613.t001

the SoSu group. During each home visit, the PTN group performed 1.3 circuits (SD: 0.5) with approximately 5.5 exercises (SD: 0.8) and 12.2 repetitions (SD: 3.9). In addition, seven participants (17%) of the PTN group performed strength circuits between home visits. Although participants of the SoSu group should not have conducted strength exercise or discussed nutritional aspects, three participants (1%) did in fact perform the circuits and talk about nutrition. When asked why, the buddies replied that they were not able to prevent the intervention.

Discussion

Our findings demonstrate that a home-based physical and nutritional intervention program carried out by trained lay volunteers significantly improves handgrip strength and physical performance of prefrail and frail community-dwelling older adults. However, it is not significantly superior to home visits with social support alone.

The increase in handgrip strength in the PTN group is comparable to changes obtained by health-care professionals conducting strength training [14, 15, 21, 22]. However, by comparing our findings to other studies it should be considered that the training protocols of our intervention and the cited interventions differed in some points. For example, in the study of Kwon et al. [14], the training was conducted only once a week and they selected different strength exercises, starting with one set and five repetitions. Our results are also comparable to changes obtained by a training program performed with a videotape [42], and it was found to be more effective than a home-based training program conducted with a booklet [43]. However, it should again be kept in mind that the intervention protocols were different.

Nevertheless, although the PTN group showed significantly increased handgrip strength, we did not find a significant difference between the PTN and SoSu group. Given this non-significant finding, the study failed to demonstrate that home visits with a physical and nutritional intervention program affect handgrip strength more than social support alone. This might be explained by the fact that the SoSu group also had a tendency towards improvement, however, with no significant increase. As we assumed that social support has comparable effects with health education or no intervention [14, 21, 43], we did not expect that high tendency, which might be traced back to the fact that the prefrail and frail community-dwelling individuals were looking forward to and prepared for the visit.

Table 2. Changes of the physical training and nutrition (PTN) and the social support (SoSu) groups from baseline to 12-week assessment.

		Within-group differences [a]		Between-group differences [b]	
	**Group**	**Mean change (95% CI)**	**p-value**	**ß (95% CI)**	**p-value**
**Handgrip strength (kg)**	PTN	2.4 (1.0–3.8)	0.001	1.3 (−0.3–2.9)	0.105
	SoSu	0.8 (−0.4–2.0)	0.189	0	
**Physical performance (score)**	PTN	1.2 (0.3–2.1)	0.009	1.0 (0.0–2.0)	0.044
	SoSu	0.5 (0.1–0.9)	0.011	0	
Balance (score)	PTN	0.4 (0.0–0.8)	<0.001	0.0 (−0.5–0.4)	0.934
	SoSu	0.5 (0.2–0.8)	0.002	0	
Gait speed (score)	PTN	0.2 (−0.2–0.6)	0.316	0.2 (−0.2–0.7)	0.231
	SoSu	−0.1 (−0.3–0.2)	0.688	0	
Lower limb muscle strength (score)	PTN	0.6 (0.2–1.0)	0.003	0.6 (0.2–1.1)	0.007
	SoSu	0.1 (−0.2–0.3)	0.464	0	
**Lean body mass (kg)**	PTN [c]	0.4 (−0.8 to 1.5)	0.546	−0.4 (−1.7–1.0)	0.606
	SoSu [d]	0.5 (−0.3–1.3)	0.235	0	
**Appendicular skeletal muscle mass (kg)**	PTN [c]	0.3 (−0.2–0.7)	0.200	0.1 (−0.5–0.6)	0.814
	SoSu [d]	0.2 (−0.1–0.5)	0.184	0	
**Appendicular skeletal muscle mass (kg/m^2)**	PTN [c]	0.1 (−0.1–0.3)	0.259	0.0 (−0.2–0.2)	0.964
	SoSu [d]	0.1 (0.0–0.2)	0.161	0	

PTN: physical training and nutrition group (n = 39); SoSu: social support group (n = 41)

[a] Differences from baseline to 12 weeks were calculated using paired t-tests.

[b] Differences between the PTN and the SoSu groups were calculated using ANCOVA for repeated measurements, adjusted for sex, age, and the corresponding baseline value, with SoSu as the reference group.

[c] n = 38, due to missing bioelectrical impedance analyses

[d] n = 35, due to missing bioelectrical impedance analyses

doi:10.1371/journal.pone.0169613.t002

Table 3. Baseline characteristics associated with an improvement in handgrip strength.

Baseline characteristics	Improvement in handgrip of ≥2.0 kg [a]	p-value
	OR (95% CI)	
**Sex**		
Female	1	
Male	1.12 (0.34–3.70)	0.849
**Age (years)**	0.96 (0.91–1.02)	0.239
**Living arrangements**		
Living alone	1	
Living with others	0.82 (0.29–2.28)	0.696
**Frailty status (SHARE-FI score)**		
Robust or prefrail	1	
Frail	2.70 (1.01–7.22)	0.048
**Nutritional status (MNA®-SF score)**		
Normal nourished	1	
Risk of malnutrition or malnourished	1.32 (0.55–3.21)	0.535
**Handgrip strength (kg)**		
≥17.9 kg in female, ≥26.5 kg in men[b]	1	
<17.9 kg in female, <26.5 kg in men[b]	0.41 (0.15–1.09)	0.073
**Physical performance (SPPB score)**	1.07 (0.91–1.25)	0.429
Balance (score)	1.17 (0.82–1.66)	0.385
Gait speed (score)	1.36 (0.88–2.10)	0.165
Lower limb muscle strength (score)	0.95 (0.65–1.38)	0.785
**BMI (kg/m^2)**	1.02 (0.92–1.13)	0.734
**Lean body mass (kg)**	1.00 (0.94–1.06)	0.887
**Appendicular skeletal muscle mass (kg)**	0.99 (0.87–1.15)	0.989
**Appendicular skeletal muscle mass (kg/m^2)**	1.01 (0.60–1.71)	0.961
**Charlson comorbidity index**	1.37 (0.99–1.89)	0.058

MMSE = Mini Mental State Examination, *SHARE-FI* = Frailty Instrument of the Survey of Health, Ageing and Retirement in Europe, *MNA®-SF* = Mini nutritional assessment-Short form, *SPPB* = Short Physical Performance Battery.

[a] The data are based on univariate logistic regression analyses with the dependent variable 'improvement in handgrip strength' (≥2.0 kg, as defined in the study protocol) [25]. Values are presented in odds ratio (OR) and 95% confidence interval (95% CI).

[b] Based on the mean values of frail individuals in the SHARE-study [29].

doi:10.1371/journal.pone.0169613.t003

The results also showed that frail subjects were more likely to improve handgrip strength than prefrail individuals. The lower starting levels and the fact that these individuals needed lower stimuli to see an improvement might be a reason. As low handgrip strength and advanced frailty status were factors associated with an improvement, it would be obvious that also physical performance was a factor associated with an increase in handgrip strength. However, this was refuted by our data. Even though, our results indicate that both weak persons and individuals with an advanced frailty status should be encouraged to conduct strength exercises in combination with a nutritional intervention.

Our intervention also affected the physical performance of the PTN and SoSu groups significantly. In turn, the increase of the PTN group was comparable to effects obtained by strength training guided by health-care professionals, both with (12,19) and without nutritional supplementation [11]. Apart from that it is remarkable that home-visits with social support alone

also significantly improved physical performance. A possible explanation for this might be that the intervention of the SoSu group affected the instrumental activities of daily living and consequently improved physical performance. This explanation is in line with recently published literature [4, 44]. Another explanation, already mentioned by Lee and colleagues [45], might be that social support influence the ability to handle frailty, as regular visits might alter personal belief, attitudes, or coping skills.

Despite these effects, no change was seen in lean body mass and appendicular skeletal muscle mass. As an increase in appendicular muscle mass is not a mandatory requirement for improving muscle strength or physical performance [46], the improvements in handgrip strength and physical performance might be explained by improved motor unit recruitment capacity and motor unit firing rate of existing skeletal muscle [1, 13]. This improvement was shown to be an early adaption to strength training, already detectable after the first week of training [47]. In that regard, our findings are comparable to those of other strength training studies which were combined with nutritional supplementation [48–50]. Only one of these other studies, with the same observational period, reported an increase in lean body mass of 1.2 kg [21]. However, this sample had higher handgrip strength and physical performance at baseline.

Considering the number of adverse events, the intervention can be considered to be safe under the given conditions (e.g. inclusion and exclusion criteria, buddies could phone healthcare professionals). Moreover, our results confirm the assumption of Bonnefoy et al. [49], who postulated that the drop-out rate in interventions supervised by trained lay-volunteers is not lower than in interventions conducted by health-care professionals.

The major strength of this study was that the intervention was carried out by trained lay-volunteers and not by health-care professionals. As the volunteers were middle-aged, conducted the home visits voluntarily, and were responsible for only one older person, a social relationship could be built up. A further strength was that the intervention was carried out at the older persons' homes. Thus, the older adults did not have to leave their home, which can be the first obstacle to attending an exercise program.

The study also has some limitations. Firstly, we did not have a control group without any home visits at all. However, this was impracticable for ethical reasons. Secondly, the intervention was a standardized program, not allowing tailoring to the particular needs of each subjects. However, tailoring would exceed the knowledge of our trained-volunteers and should be reserved to health-care professionals. Thirdly, due to organizational matters, blinding was not realizable (e.g. study participants called the study team whenever questions arose). Fourthly, the effects on nutritional aspects are not shown in the present publication. However, a further manuscript published as part of this study showed that the MNA®-LF score increased significantly by 8% in the PTN and by 5% in the SoSu group [51]. Finally, the compliance to the intervention was slightly different to the study protocol (e.g. fewer than two circuits with six exercises were performed, and it was found that the prefrail and frail persons did not conduct strength exercises alone between the home visits).

Further research will show, if the achieved effects can be maintained after 12 weeks. Additionally, another paper will examine if these home visits also affect the health status of the buddies. For similar future studies, we consider hospitals to be the wrong setting for recruiting participants, as the majority of these individuals did not fulfill the applied inclusion criteria, and many of the eligible individuals declined participation.

Conclusion

Handgrip strength in the intervention group increased, by a clinically relevant value, and this effect is comparable to that obtained by health-care professionals. Therefore, home visits with

a physical and nutritional intervention program provided by volunteers could offer a new perspective in the care of community-dwelling older persons. Notably, in this sample social support also plays an important role. Due to this fact, we did not find a significant difference between home visits with a physical and nutritional intervention program and home visits with social support only. Further results indicate that also individuals with an advanced frailty status should be encouraged to do strength exercises in combination with a nutritional intervention.

Supporting Information

S1 File. Dataset. Dataset from the Medical University of Vienna.
(SAV)

S2 File. Consort checklist.
(DOC)

S3 File. Study protocol.
(PDF)

Acknowledgments

We would like to thank Martin Oberbauer, Melissa Vallant, Sophia Koepruner, Lisa Buchinger, Lisa Penold, and Michaele Ehrenberger for their assistance in conducting the study, Georg Heinze for the support in statistical issues, and Mark Ackerley for the professional proofreading.

Author Contributions

Conceptualization: TED KS.

Data curation: EL SH AK.

Formal analysis: SH TED EL.

Funding acquisition: TED KS.

Investigation: SH TED EL AK CL KS.

Methodology: TED KS CL EL SH AK.

Project administration: TED EL.

Supervision: KS TED ST.

Visualization: SH.

Writing – original draft: SH.

Writing – review & editing: TED KS CL EL AK ST.

References

1. Cadore EL, Pinto RS, Bottaro M, Izquierdo M. Strength and endurance training prescription in healthy and frail elderly. Aging Dis 2014; 5(3):183–95. PubMed Central PMCID: PMC4037310. doi: 10.14336/AD.2014.0500183 PMID: 24900941

2. Fried LP, Ferrucci L, Darer J, Williamson JD, Anderson G. Untangling the concepts of disability, frailty, and comorbidity: implications for improved targeting and care. J Gerontol A Biol Sci Med Sci. 2004; 59(3):255–63. Epub 2004/03/20. PMID: 15031310

3. Espinoza SE, Fried LP. Risk Factors for Frailty in the Older Adult. Clin Geriatr. 2007; 15:37–44.
4. Ng TP, Feng L, Nyunt MS, Feng L, Niti M, Tan BY, et al. Nutritional, Physical, Cognitive, and Combination Interventions and Frailty Reversal Among Older Adults: A Randomized Controlled Trial. Am J Med. 2015; 128(11):1225–36 e1. doi: 10.1016/j.amjmed.2015.06.017 PMID: 26159634
5. Rolland Y, Dupuy C, Abellan van Kan G, Gillette S, Vellas B. Treatment strategies for sarcopenia and frailty. Med Clin North Am. 2011; 95(3):427–38, ix. Epub 2011/05/10. doi: 10.1016/j.mcna.2011.02.008 PMID: 21549870
6. Norman K, Stobaus N, Gonzalez MC, Schulzke JD, Pirlich M. Hand grip strength: outcome predictor and marker of nutritional status. Clin Nutr. 2011; 30(2):135–42. doi: 10.1016/j.clnu.2010.09.010 PMID: 21035927
7. Romero-Arenas S, Martinez-Pascual M, Alcaraz PE. Impact of resistance circuit training on neuromuscular, cardiorespiratory and body composition adaptations in the elderly. Aging Dis. 2013; 4(5):256–63. PubMed Central PMCID: PMC3794722. doi: 10.14336/AD.2013.0400256 PMID: 24124631
8. Fried LP, Tangen CM, Walston J, Newman AB, Hirsch C, Gottdiener J, et al. Frailty in older adults: evidence for a phenotype. J Gerontol A Biol Sci Med Sci. 2001; 56(3):M146–56. Epub 2001/03/17. PMID: 11253156
9. Bonnefoy M, Berrut G, Lesourd B, Ferry M, Gilbert T, Guerin O, et al. Frailty and nutrition: searching for evidence. J Nutr Health Aging. 2015; 19(3):250–7. doi: 10.1007/s12603-014-0568-3 PMID: 25732208
10. Bauer J, Biolo G, Cederholm T, Cesari M, Cruz-Jentoft AJ, Morley JE, et al. Evidence-based recommendations for optimal dietary protein intake in older people: a position paper from the PROT-AGE Study Group. J Am Med Dir Assoc 2013; 14(8):542–59. doi: 10.1016/j.jamda.2013.05.021 PMID: 23867520
11. Zech A, Drey M, Freiberger E, Hentschke C, Bauer JM, Sieber CC, et al. Residual effects of muscle strength and muscle power training and detraining on physical function in community-dwelling prefrail older adults: a randomized controlled trial. BMC Geriatr. 2012; 12:68. Epub 2012/11/09. PubMed Central PMCID: PMCPMC3538686. doi: 10.1186/1471-2318-12-68 PMID: 23134737
12. Cadore EL, Rodriguez-Manas L, Sinclair A, Izquierdo M. Effects of different exercise interventions on risk of falls, gait ability, and balance in physically frail older adults: a systematic review. Rejuvenation Res 2013; 16(2):105–14. PubMed Central PMCID: PMC3634155. doi: 10.1089/rej.2012.1397 PMID: 23327448
13. Fiatarone MA, O'Neill EF, Ryan ND, Clements KM, Solares GR, Nelson ME, et al. Exercise training and nutritional supplementation for physical frailty in very elderly people. N Engl J Med 1994; 330(25):1769–75. Epub 1994/06/23. doi: 10.1056/NEJM199406233302501 PMID: 8190152
14. Kwon J, Yoshida Y, Yoshida H, Kim H, Suzuki T, Lee Y. Effects of a combined physical training and nutrition intervention on physical performance and health-related quality of life in prefrail older women living in the community: a randomized controlled trial. J Am Med Dir Assoc 2015; 16(3):263 e1–8. Epub 2015/02/11.
15. Abizanda P, Lopez MD, Garcia VP, Estrella JD, da Silva Gonzalez A, Vilardell NB, et al. Effects of an Oral Nutritional Supplementation Plus Physical Exercise Intervention on the Physical Function, Nutritional Status, and Quality of Life in Frail Institutionalized Older Adults: The ACTIVNES Study. J Am Med Dir Assoc 2015; 16(5):439.
16. Buman MP, Giacobbi PR Jr., Dzierzewski JM, Aiken Morgan A, McCrae CS, Roberts BL, et al. Peer volunteers improve long-term maintenance of physical activity with older adults: a randomized controlled trial. J Phys Act Health 2011; 8 Suppl 2:257–66. Epub 2011/09/23. PubMed Central PMCID: PMCPMC3181088.
17. French DP, Olander EK, Chisholm A, Mc Sharry J. Which behaviour change techniques are most effective at increasing older adults' self-efficacy and physical activity behaviour? A systematic review. Ann Behav Med 2014; 48(2):225–34. doi: 10.1007/s12160-014-9593-z PMID: 24648017
18. Ginis KA, Nigg CR, Smith AL. Peer-delivered physical activity interventions: an overlooked opportunity for physical activity promotion. Transl Behav Med. 2013; 3(4):434–43. PubMed Central PMCID: PMC3830020. doi: 10.1007/s13142-013-0215-2 PMID: 24294332
19. Payette H, Boutier V, Coulombe C, Gray-Donald K. Benefits of Nutritional Supplementation in Free-living, Frail, Undernourished Elderly People. J Am Diet Assoc 2002; 102(8):1088–95. PMID: 12171453
20. Milne AC, Potter J, Vivanti A, Avenell A. Protein and energy supplementation in elderly people at risk from malnutrition. Cochrane Database Syst Rev. 2009;(2):CD003288. Epub 2009/04/17. doi: 10.1002/14651858.CD003288.pub3 PMID: 19370584
21. Tieland M, Dirks ML, van der Zwaluw N, Verdijk LB, van de Rest O, de Groot LC, et al. Protein supplementation increases muscle mass gain during prolonged resistance-type exercise training in frail elderly people: a randomized, double-blind, placebo-controlled trial. J Am Med Dir Assoc 2012; 13(8):713–9. Epub 2012/07/10. doi: 10.1016/j.jamda.2012.05.020 PMID: 22770932

22. Oesen S, Halper B, Hofmann M, Jandrasits W, Franzke B, Strasser EM, et al. Effects of elastic band resistance training and nutritional supplementation on physical performance of institutionalised elderly—A randomized controlled trial. Exp Gerontol 2015; 72:99–108. doi: 10.1016/j.exger.2015.08.013 PMID: 26341720
23. Schilp J, Kruizenga HM, Wijnhoven HA, van Binsbergen JJ, Visser M. Effects of a dietetic treatment in older, undernourished, community-dwelling individuals in primary care: a randomized controlled trial. Eur J Nutr 2013; 52(8):1939–48. doi: 10.1007/s00394-013-0495-9 PMID: 23361389
24. Buckinx F, Rolland Y, Reginster J-Y, Ricour C, Petermans J, Bruyère O. Burden of frailty in the elderly population: perspectives for a public health challenge. Arch Public Health 2015; 73(1):19. doi: 10.1186/s13690-015-0068-x PMID: 25866625
25. Dorner TE, Lackinger C, Haider S, Luger E, Kapan A, Luger M, et al. Nutritional intervention and physical training in malnourished frail community-dwelling elderly persons carried out by trained lay "buddies": study protocol of a randomized controlled trial. BMC Public Health. 2013; 13(1):1232.
26. Randomizer for clinical trials 1.8.1. Available from: www.meduniwien.ac.at/randomizer/web/login.php.
27. Dorner TE, Luger E, Tschinderle J, Stein KV, Haider S, Kapan A, et al. Association between nutritional status (MNA®-SF) and frailty (SHARE-FI) in acute hospitalised elderly patients. J Nutr Health Aging. 2014; 18(3):264–9. doi: 10.1007/s12603-013-0406-z PMID: 24626753
28. Dale O, Salo M. The Helsinki Declaration, research guidelines and regulations: present and future editorial aspects. Acta Anaesthesiol Scand. 1996; 40(7):771–2. Epub 1996/08/01. PMID: 8874560
29. Romero-Ortuno R, Walsh CD, Lawlor BA, Kenny RA. A frailty instrument for primary care: findings from the Survey of Health, Ageing and Retirement in Europe (SHARE). BMC Geriatr. 2010; 10:57. Epub 2010/08/25. PubMed Central PMCID: PMC2939541. doi: 10.1186/1471-2318-10-57 PMID: 20731877
30. Kaiser MJ, Bauer JM, Ramsch C, Uter W, Guigoz Y, Cederholm T, et al. Validation of the Mini Nutritional Assessment short-form (MNA-SF): a practical tool for identification of nutritional status. J Nutr Health Aging 2009; 13(9):782–8. Epub 2009/10/09. PMID: 19812868
31. Folstein MF, Folstein SE, McHugh PR. "Mini-mental state". A practical method for grading the cognitive state of patients for the clinician. J Psychiatr Res. 1975; 12(3):189–98. Epub 1975/11/01. PMID: 1202204
32. University of Harvard. Healthy eating plate. [19 July 2013]. Available from: www.hsph.harvard.edu/nutritionsource/healthy-eating-plate/.
33. Charlson ME, Pompei P, Ales KL, MacKenzie CR. A new method of classifying prognostic comorbidity in longitudinal studies: development and validation. J Chronic Dis. 1987; 40(5):373–83. Epub 1987/01/01. PMID: 3558716
34. Roberts HC, Denison HJ, Martin HJ, Patel HP, Syddall H, Cooper C, et al. A review of the measurement of grip strength in clinical and epidemiological studies: towards a standardised approach. Age Ageing. 2011; 40(4):423–9. doi: 10.1093/ageing/afr051 PMID: 21624928
35. Guralnik JM, Simonsick EM, Ferrucci L, Glynn RJ, Berkman LF, Blazer DG, et al. A short physical performance battery assessing lower extremity function: association with self-reported disability and prediction of mortality and nursing home admission. J Gerontol 1994; 49(2):M85–94. Epub 1994/03/01. PMID: 8126356
36. Data Input. Das B.I.A.-Kompendium (3. edition) Darmstadt2009 [19. July 2016]. Available from: www.datainput.de/media/pdf-deutsch/Kompendium_III_Ausgabe_2009.pdf.
37. Pace N, Rathbun EN. Studies on body composition: III. The body water and chemically combined nitrogen content in relation to fat content. J Biol Chem. 1945; 158:685–91.
38. Sergi G, De Rui M, Veronese N, Bolzetta F, Berton L, Carraro S, et al. Assessing appendicular skeletal muscle mass with bioelectrical impedance analysis in free-living Caucasian older adults. Clin Nutr. 2015; 34(4):667–73. doi: 10.1016/j.clnu.2014.07.010 PMID: 25103151
39. Streiner D, Geddes J. Intention to treat analysis in clinical trials when there are missing data. Evid Based Ment Health. 2001; 4(3):70–1. Epub 2002/05/15. PMID: 12004740
40. Dodds RM, Syddall HE, Cooper R, Benzeval M, Deary IJ, Dennison EM, et al. Grip strength across the life course: normative data from twelve British studies. PloS one. 2014; 9(12):e113637. PubMed Central PMCID: PMC4256164. doi: 10.1371/journal.pone.0113637 PMID: 25474696
41. Zhang S, Paul J, Nantha-Aree M, Buckley N, Shahzad U, Cheng J, et al. Empirical comparison of four baseline covariate adjustment methods in analysis of continuous outcomes in randomized controlled trials. Clin Epidemiol 2014; 6:227–35. PubMed Central PMCID: PMC4105274. doi: 10.2147/CLEP.S56554 PMID: 25053894
42. Vestergaard S, Kronborg C, Puggaard L. Home-based video exercise intervention for community-dwelling frail older women: a randomized controlled trial. Aging Clin Exp Res 2008; 20(5):479–86. Epub 2008/11/29. PMID: 19039291

43. Nelson ME, Layne JE, Bernstein MJ, Nuernberger A, Castaneda C, Kaliton D, et al. The effects of multidimensional home-based exercise on functional performance in elderly people. J Gerontol A Biol Sci Med Sci. 2004; 59(2):154–60. Epub 2004/03/05. PMID: 14999030

44. Willis SL, Tennstedt SL, Marsiske M, Ball K, Elias J, Koepke KM, et al. Long-term effects of cognitive training on everyday functional outcomes in older adults. JAMA 2006; 296(23):2805–14. PubMed Central PMCID: PMC2910591. doi: 10.1001/jama.296.23.2805 PMID: 17179457

45. Lee WJ, Chen LK, Peng LN, Chiou ST, Chou P. Personal mastery attenuates the adverse effect of frailty on declines in physical function of older people: A 6-year population-based cohort study. Medicine. 2016; 95(34):e4661. Epub 2016/08/26. doi: 10.1097/MD.0000000000004661 PMID: 27559969

46. McGregor RA, Cameron-Smith D, Poppitt SD. It is not just muscle mass: a review of muscle quality, composition and metabolism during ageing as determinants of muscle function and mobility in later life. Longev Healthspan. 2014; 3(1):9. Epub 2014/12/19. PubMed Central PMCID: PMCPMC4268803. doi: 10.1186/2046-2395-3-9 PMID: 25520782

47. Kamen G, Knight CA. Training-related adaptations in motor unit discharge rate in young and older adults. J Gerontol A Biol Sci Med Sci. 2004; 59(12):1334–8. Epub 2005/02/09. PMID: 15699535

48. Kim HK, Suzuki T, Saito K, Yoshida H, Kobayashi H, Kato H, et al. Effects of exercise and amino acid supplementation on body composition and physical function in community-dwelling elderly Japanese sarcopenic women: a randomized controlled trial. J Am Geriatr Soc. 2012; 60(1):16–23. Epub 2011/12/07. doi: 10.1111/j.1532-5415.2011.03776.x PMID: 22142410

49. Bonnefoy M, Cornu C, Normand S, Boutitie F, Bugnard F, Rahmani A, et al. The effects of exercise and protein-energy supplements on body composition and muscle function in frail elderly individuals: a long-term controlled randomised study. Br J Nutr 2003; 89(5):731–9. Epub 2003/05/02. doi: 10.1079/BJN2003836 PMID: 12720593

50. Sullivan DH, Roberson PK, Smith ES, Price JA, Bopp MM. Effects of muscle strength training and megestrol acetate on strength, muscle mass, and function in frail older people. J Am Geriatr Soc. 2007; 55(1):20–8. Epub 2007/01/20. doi: 10.1111/j.1532-5415.2006.01010.x PMID: 17233681

51. Luger E, Dorner TE, Haider S, Kapan A, Lackinger C, Schindler K. Effects of a Home-Based and Volunteer-Administered Physical Training, Nutritional, and Social Support Program on Malnutrition and Frailty in Older Persons: A Randomized Controlled Trial. J Am Med Dir Assoc 2016; 17(7):671 e9–e16. Epub 2016/06/28.
