# Supplementary material for: Change in inflammatory parameters in prefrail and frail persons obtaining physical training and nutritional support provided by lay volunteers: A randomized controlled trial
Source: PLoS One. 2017 Oct 12;12(10):e0185879. doi: 10.1371/journal.pone.0185879 (PMC5638289; doi:10.1371/journal.pone.0185879)
Supplement: S4 File — (PDF) [file pone.0185879.s005.pdf]

Nutrition intervention and physical training in malnourished frail community-dwelling elderly subjects carried out by trained lay “buddies”

Principal Investigator: Ass.-Prof. Priv.-Doz. Dr. med. univ. Thomas E. Dorner, MPH [1]
Univ.-Lektor Priv.-Doz. Dr. Karin E. Schindler [2]

Co-Investigators: Mag. Dr. Christian Lackinger [3]
Dr. Eva Schwarz, MPH [1]
Eva Luger, Bakk. MSc. [1]
Mag. Sandra Haider, Bakk. [1, 3]
Mag. Ali Kapan [1]

[1] Institute of Social Medicine, Centre for Public Health, **Medical University of Vienna**

[2] Department of Medicine III, Division of Endocrinology and Metabolism, **Medical University of Vienna**

[3] Department for Preventive Medicine, **SPORTUNION Austria, Vienna**

Contact:

Ass.-Prof. Priv.-Doz. Dr. med. univ. Thomas E. Dorner, MPH

Telephone: +431-40160-34884

E-Mail: thomas.dorner@meduniwien.ac.at

Univ.-Lektor Priv.-Doz. Dr. Karin E. Schindler

Telephone: +431-40400-2032

E-Mail: karin.schindler@meduniwien.ac.at

Contents

1. Background 4

2. Study objectives 6

2.1 Research question, operational hypotheses 6

3. Methodological approaches 8

3.1 Population 8

3.1.1 Recruitment 11

3.1.2 Sample size calculation 12

3.1.3 Intervention 13

3.1 Study design 16

3.2 Major parameter 16

3.2.1 Primary endpoint 16

3.2.2 Secondary endpoints 16

3.3 Statistical Analysis 22

4. Financial Frame 23

5. Insurance 23

6. Risk and benefits 23

7. Measuring Project success 24

8. Workplan 25

9. Regulatory framework 27

9.1 Data administration 27

9.2 Ethical and legal aspects 28

9.3 Patients' informed consent 28

9.4 Documentation of the study results 28

9.5 Intended use of the data 28

9.6 Protocol changes and amendments 28

10. References 29

1. Background

In elderly subjects nutrition-related problems are very common and can lead to serious health hazards, increase mortality, morbidity, dependency and institutionalization, and reduce quality of life [1-6]. Malnutrition is defined as a chronic state in which a combination of varying degrees of over- and undernutrition and inflammatory activity changes body composition [7, 8].

In elderly subjects, malnutrition (under- and overnutrition) is also associated with frailty and participates to the pathogenesis of frailty [9-12]. Besides hormonal changes and inflammatory processes, nutrition plays a decisive role in the development of frailty. Weight loss is one of the defining characteristics of frailty [13]. Frailty is considered as a state of high vulnerability for adverse health outcomes, including disability, dependency, falls, need for long-term care and mortality [14]. Thus, people suffering from frailty are at higher risk for mortality and morbidity, dependency, hospitalization, falls, disability, social isolation and they have a decrease in quality of life [15]. Besides of malnutrition, sarcopenia (loss of muscle mass), low physical activity, low exercise capacity and muscle strength, chronic inflammation, and exhaustion (both, physical and mental) are facts that greatly contribute to the development of frailty [10]. Consequences of exhaustion like social retreat and isolation enhance the factors malnutrition and low physical activity level and therefore the risk for frailty.

In Europe, prevalence rate of frailty, according to the SHARE-FI criteria [16], in subjects aged 65 years and older is 17.0% and additional 42.3% are prefrail, an intermediate state between robust and frail with a higher risk to develop frailty. In Austria, prevalence rates for frailty and pre-frailty are 10.8% and 40.7%, respectively [17]. Based on these findings, in the year 2009 a number of 217.000 subjects affected from frailty and 1 million subjects affected from pre-frailty can be calculated in Austria. Through extrapolation of the demographic development only, for the year 2050 a number of 356.000 frail and 1.5 million pre-frail subjects can be expected [18]. Hence, in elderly subjects malnutrition, sarcopenia and frailty are due to demographic trends one of the greatest challenges to the health care system and to society.

It has been shown that frail older adults have a low intake of energy, protein and several micronutrients [19] and also lower plasma concentrations of various nutrients compared with nonfrail individuals [20, 21]. According to the Austrian Nutrition Report, only two thirds of subjects aged 65 years and older (Austrian population) reach the recommended amount of energy. The mean protein uptake in the general elderly population is with 15 energy percent in the recommended range. However, in institutionalized elderly subjects, 45% do not reach

the recommended protein uptake of 0.8 g/kg body weight [22]. Since the nutrition status often improves in subjects after nursing home admission [23], it can be concluded, that energy and especially protein uptake for elderly must be especially improved in frail subjects who are still living at home. Additionally, a healthy dietary pattern is associated with a lower risk of being frail [24].

Physical inactivity is one of the main risk factors of frailty. Decrease in the function of the cardiovascular system and loss of muscle mass and muscle strength are severe conditions in elderly people. General physical activity leads primary to energy-expenditure. In contrast, exercise training (booth – endurance and strength training) leads to significant improvements in fitness levels and increase the muscle mass. Recent research has shown that strength training is a powerful tool in order to improve health status and quality of life in elderly people (insulin sensitivity, pain, memory, prevention of falls) [25, 26]. A further important benefit for elderly subjects who perform strength training is the empowerment of people to remain autonomous and independent and therefore nursing home admission can be postponed. In contrast to endurance training, strength training has several advantages: people with impairments of the musculoskeletal system can also do a large number of strength training exercises. Strength training can easily become a part of daily routine. After a period of supervised training, effects can be kept by self-conducted exercises. Home-based training intervention with a video has been shown to be effective to improve muscle strength in frail elderly women [27].

A well-structured nutritional intervention program in combination with strength training is the most effective therapeutic option for the treatment of frailty [28]. Through this intervention many negative outcomes of malnourished frail people like morbidity and risk of falls can be reduced. Additionally, general well-being, quality of life, muscle strength, and activities of daily living can be improved [29].

In conclusion, scientific literature in this field gives evidence that…

- …there is an interrelation between malnutrition, frailty and chronic inflammation.
- …malnutrition and frailty lead to a variety of adverse health outcomes including dependency and institutionalization and therefore frailty is one of the most relevant health problems in elderly subjects.
- …adverse outcomes which are associated with malnutrition and frailty are preventable through nutrition intervention and structured muscle training.

There is a lack of evidence regarding…

- …the impact of nutrition and training interventions in malnourished frail elderly subjects on various health outcomes like quality of life, hospitalization rates, health resources like social networks, etc.
- …the impact on the health status of such an intervention on lay persons, who perform the intervention together with the malnourished, frail subjects.
- …the optimal structure and setting of such an intervention as community dwellers (most studies in this field have been performed in institutionalized subjects).

2. Study objectives

The aim of the proposed study is to improve the nutrition status, to increase muscle strength, and to improve the overall health status in malnourished, frail or pre-frail community-dwelling elderly persons. For that purpose it engages elderly, lay, fit (robust) subjects who work voluntarily as buddies for malnourished (pre)frail adults with regards to their nutritional status and physical activity. These buddies will visit the malnourished (pre)frail subjects at home twice a week, take care of their food storage, talk about nutrition and nutrition-related problems with the help of a portfolio, and play a nutrition-related game. Additionally the buddies will perform a strength training together with the frail subjects with the help of a DVD, video or with a training portfolio. In this project, the health status of both (frail malnourished subjects and buddies) is expected to improve, due to the active engagement with nutrition and physical training. Due to the project the social networks and social interaction of both parties will be strengthened. In Addition, health resources and quality of life will be increased. A clear objective of this study is to perform the nutrition and training intervention in frail people’s home environment, not in an institutionalized setting.

2.1 Research question, operational hypotheses

Malnourished (pre)frail community-dwelling adults benefit more from the home based nutritional and physical intervention program carried out by buddies, than their counterparts with merely regular home visits.

Operational hypotheses:

- **Main hypothesis**
  - **H_0:** A structured training and nutrition intervention yields in no significant difference in handgrip strength (dynamometer) in the intervention group (malnourished frail adults) compared to the control group.
  - **H_1:** A structured training and nutrition intervention yields in significant higher handgrip strength (dynamometer) in the intervention group (malnourished frail adults) compared to the control group.

- **Secondary hypotheses**
  - *Buddies and malnourished frail adults:*
    Following parameters increases to a greater degree in the intervention group than in the control group:
    - Nutritional status (MNA-SF for screening and MNA-LF for assessment for malnutrition)
    - Quality of life (WHOQOL-BREF/OLD)
    - Social support (F-SozU)
    - Quality of the whole diet (MEDIAS + Protein-FFQ)
    - Anthropometry (weight, BMI) and body composition (BIA)
    - Muscle strength (MASS, Concept 2 dyno)
    - Sings of malnutrition and frailty (laboratory parameters)
    - Amount of physical activity (PASE)
  - *Malnourished frail adults:*
    Following parameters decreases to a greater degree in the intervention group than in the control group:
    - Frequency of hospitalization
    - Frequency of falls

    Following parameters increases to a greater degree in the intervention group than in the control group:
    - Frailty status (SHARE-FI)
    - Cognitive function (MMSE)

- Balance and mobility components (SPPB score)
- Fear of falling (FES-I)
- Qualitative and quantitative contents of refrigerators (predefined protocol and picture of refrigerator)

o *Buddies:*
Following parameters increases to a greater degree in the intervention group than in the control group:

- Daily physical activity (pedometer)

3. Methodological approaches

3.1 Population

Malnourished, (pre)frail elderly subjects as well as buddies are randomly assigned to an intervention or a control group for the first 10-12 weeks. For all involved subjects, malnourished, (pre)frail elderly subjects and buddies in either group, at the beginning (visit 1), after 10-12 weeks (visit 2), after 6 months (visit 3) and after 12 months (visit 4) different measures are assessed.
For malnourished (pre)frail subjects health-, nutritional status (MNA), frailty (SHARE-FI), quality of life (WHOQOL/-OLD), social support (F-SozU), quality of whole diet (MEDIAS and Protein-FFQ), qualitative and quantitative contents of refrigerators (predefined protocol and picture of refrigerator), amount of physical activity (PASE), cognitive function (MMSE), balance and mobility (SPPB), fear of falling (FES-I), anthropometry (weight, BMI), body composition (BIA), muscle strength (MASS and concept 2 dyno), signs of malnutrition and frailty (laboratory parameters) and refrigerator content (protocol) will be performed three times. Diagnoses according to the hospital discharge letter at the beginning of the study will be recorded, and incidental medical conditions (falls, hospitalization) during these 6 months will be documented.
For buddies health status, quality of life (WHOQOL), social support (F-SozU), quality of whole diet (MEDIAS and Protein-FFQ), amount of physical activity (PASE), daily physical activity (pedometer), anthropometry (weight, BMI), body composition (BIA), muscle strength (MASS and concept 2 dyno), grip strength (dynamometer) and signs of malnutrition and frailty (laboratory parameters) will be performed three times.
Table 3.1 shows the measurements at several times (visit 1, 2, 3, 4) in malnourished (pre)frail elderly subjects and buddies.

Measurements of health status

- Self-rated health
- *Quality of life* assessed by the World Health Organization Quality of Life WHOQOL-BREF and by 3 dimensions of the WHOQOL-OLD ("sensory functions", "autonomy", "activities in the past, present and future")
- Social support measured with the short version of the questionnaire F-SozU (“Fragebogen zur Sozialen Unterstützung”)
- Frequency of hospitalization (in malnourished, (pre)frail elderly subjects only)
- Frequency of falls (in malnourished, (pre)frail elderly subjects only)
- Documentation of fear of falling with the FES-I (Falls Efficacy Scale-International Version) in older person (in malnourished, (pre)frail elderly subjects only)

Measurements of nutrition status, physical activity and frailty

- Nutritional status assessed by the MNA (Mini Nutritional Assessment)
- Frailty status assessed with the SHARE-FI (Frailty Instrument for Primary Care of the Survey of Health, Ageing and Retirement in Europe)
- Cognitive function with the MMSE (Mini Mental State Examination) (in malnourished, (pre)frail elderly subjects only)
- Body mass index (BMI)
- Body composition assessed with the BIA (Bioelectrical Impedance Analysis)
- *Quality of the diet* assessed by the Mediterranean Diet Adherence Screener (MEDIAS) a food frequency questionnaire assessing the protein intake and questions concerning supplements
- Qualitative and quantitative contents of refrigerators (predefined protocol of the refrigerator) (in malnourished, (pre)frail elderly subjects only)
- Muscle strength assessed with the MASS (Measurement of age and sex related reference values of muscle strength) and the Concept dyno 2.
- Daily physical activity assessed with pedometer and PASE (Physical Activity Scale for the elderly)
- Balance and mobility components assessed with the SPPB (Short Physical Performance Battery) (in malnourished, (pre)frail elderly subjects only)
- Laboratory findings for malnutrition or frailty (albumin, total cholesterol, transferrin, triglyceride, 25-OH-VitD3, folic acid, CRP, IL-6, TNF-Alpha, leucocytes)

Table 3.1 Measurements at several times in malnourished, (pre)frail elderly subjects (F) and buddies (B)

	Selection	Visit 1 (time 0)		Visit 2 (after 10-12 weeks)		Visit 3 (after 6 months)		Visit 4 (after 12 months)	
	F	F	B	F	B	F	B	F	B
Long-term medication	X								
Comorbidities	X								
WHOQOL		X	X	X	X	X	X		
WHOQOL-OLD		X		X		X			
F-SozU		X	X	X	X	X	X		
Frequency of hospitalization		X		X		X			
Frequency of falls		X		X		X			
FES-I		X		X		X			
MNA-SF	X								
MNA-LF		(X)		X		X		X	
SHARE-FI	X			X		X		X	
MMSE	X			X		X		X	
BIA		X	X	X	X	X	X	X	X
Anthropometry	X		X	X	X	X	X		
Food Frequency Questionnaire (Proteins) and Supplements		X	X	X	X	X	X		
MEDIAS		X	X	X	X	X	X		
Protocol of the refrigerator content		X		X		X			
MASS		X	X	X	X	X	X		
Concept dyno 2		X	X	X	X	X	X		
Pedometer			X		X		X		
Modified PASE and FEG		X	X	X	X	X	X		
SPPB		X		X		X			
Handgrip (dynamometer)	X		X	X	X	X	X		
Laboratory parameters		X	X	X	X	X	X		
Personal information		X	X						
Expectations/wishes of the intervention		X	X						
Appraisal of the intervention				X	X	X	X		
Questions about continued visits								X	X

F = malnourished (pre)frail elderly persons; B = Buddies
MNA-SF = Mini nutritional assessment Short-Form; MNA-LF = Mini nutritional assessment Long-Form; SHARE-FI = Frailty Instrument for Primary Care of the Survey of Health, Ageing and Retirement in Europe; MMSE = Mini-Mental State Examination; WHOQOL = World Health Organization Quality of Life; F-SozU = Fragebogen zur sozialen Unterstützung; MEDIAS = Mediterranean Diet Adherence Screener, FFQ = Food Frequency Questionnaire; BIA = Bioelectrical Impedance Analysis; SPPB = Short Physical Performance Battery; MASS = Measurement of age and sex related reference values of muscle strength; FES-I = Falls Efficacy Scale International; PASE = Physical Activity Scale for the Elderly;

3.1.1 Recruitment

Malnourished (pre)frail persons:

As it was done in the prestudy "Frailty, nutritional status and readiness for training and nutritional intervention in elderly people in Vienna hospitals" [30-32], malnourished (pre)frail subjects 65 years and older, who were inpatients in acute hospitals in Vienna (AKH), wards for internal medicine and close to discharge will be recruited.

Inclusion criteria for malnourished, (pre)frail elderly subjects:

- Age 65 years or older
- Living in Vienna
- Community dwelling
- No medical contraindication for the performance of a physical strength training (no "No" to the question 'Has your recently doctor told you not to exercise?")
- Have to be able to walk (with or without a walking aid)
- Malnutrition or at risk of malnutrition according to the MNA-SF (Screening: 0-11 points) OR Frail or pre-frail according to the SHARE-FI (female: >2.130; male: >3.005)
- Capability to consent

Exclusion criteria for malnourished, (pre)frail elderly subjects:

- Age younger than 65 years
- Admission to nursing home planned
- Nursing level ("Pflegestufe") 6 or 7
- Chemotherapy and/or radiotherapy currently or planned
- Insulin treated diabetes mellitus in the medical history
- COPD Stadium III or IV
- Dialysis patient or chronic renal insufficiency with protein restriction
- Cannot understand the German language
- Severe impaired cognitive function according to the MMSE (≤17 points)

Buddies:

Buddies will be recruited and trained in cooperation with the "Wiener Hilfswerk" in standardized procedures. This organization already offers routinely volunteer home visits for

elderly subjects. This is one of the largest organisations in Austria offering care-giving services for elderly and sick persons (http://www.hilfswerk.at/english; 25.5.2012).

Inclusion criteria for buddies:

- Age 50 years or older
- Signed informed consent
- Readiness to participate as buddy in principle both for intervention group and control group (for the first 10-12 weeks)
- Commitment to keep to the protocol for at least 6 months

3.1.2 Sample size calculation

For sample size calculation, the difference in handgrip strength (from baseline to 10-12 weeks) will be considered as a clinical marker of muscle function. We examine its relationships with diet [24, 33] and physical activity [34] in a community-dwelling cohort. Given a clinically relevance difference of 2 kg in handgrip strength (intervention group improves by 2 kg more than the control group in handgrip strength), a standard deviation of 3 kg of the differences, a two-sided significance level of 0.05, a sample size of n=36 per group is needed to reach 80% statistical power. Since imputation for drop-outs may have some unestimable effect on the assumed standard deviation of the differences, the sample size is increased to n=40 per group.

To reach this sample size, a total of 120 buddies will be recruited in the main phase of the study, in 4 waves with a time interval of 4 months. With this amount of buddies it is guaranteed that 40 buddies can withdraw their consent to function as buddy.

- Goal criterion handgrip strength:
    - Expected value μ_1 (intervention) = 16 kg
      μ_2 (control) = 14 kg (based on the results of the prestudy [30, 32])
    - Standard deviation σ = 3 kg
    - Difference $\delta = \mu 1 - \mu 2$ = 2 kg
- Two-sample t test
- Significance level α = 0.05
- Power $1-\beta$ = 0.80

3.1.3 Intervention

Control group

In the first phase for the study participants (first 10-12 weeks) subjects will be divided randomly into two groups: An intervention group and a control group. Participants in the control group will be visited regularly by buddies, but they do not specifically monitor the nutritional status or perform physical training in these first 10-12 weeks. Instead of that, buddies will be provided with a portfolio of possible activities they could perform together with the study participants (e.g. specific cognition training). After this first phase of 10-12 weeks, nutrition and strength training intervention will be performed also with the control group. 8-10 weeks are considered to be satisfying long enough to observe effects in the intervention group compared to a control group [25, 26]. The interval was chosen as short as possible that subjects in the training group who have already agreed to participate in the nutrition and training intervention and looking forward to it, are not waiting long to gain the benefits of this intervention.

Intervention group

Home visits of the buddies at the home of the malnourished (pre)frail elderly subjects will take place twice a week and last for approximately one hour.

Nutritional intervention

The aim of the nutritional intervention is to obtain adequate protein, energy and other nutrient intake, preferable by regular foods and beverages. This intervention will include an in-depth education program with general information about healthy eating habits, which emphasizes an increased intake of fruits and vegetables, protein-rich foods and fluids.

Buddies will monitor the nutritional status of their clients with regards to adequate food storage in the household, food hygiene (e.g. no expired or moldy food), food with a satisfying amount of calories, proteins, and of fluids. A sufficient protein intake will be guaranteed by the amount of dairy products and legumes in the household.

During home visits, buddies will reflect the patients’ diet since the last visit. For this purpose, buddies will be equipped with a portfolio with nutrient dense recipes and photographs of high-protein dishes. Furthermore, in this portfolio there will be tables in which the amount of proteins of common foods can be seen.

Buddies will be trained to observe patients for signs of dehydration and rapid weight loss, which would require further medical attention. Additionally, they will be instructed to pay attention to nutritional risk factors.

Buddies will be equipped with a check list respectively education book with all required nutritional aspects such as the importance of healthy nutrition at any age, health benefits of

eating more fruits and vegetables, the importance of protein-rich foods and risk factors for malnutrition, the importance of choosing a variety of foods, tips about grocery shopping, meal time, frequency of foods and drinks, portion size and cooking methods. The nutritional advice will be to eat three main dishes and 2-3 between-meal snacks including meat, fish or egg, fruit and vegetables, dairy products and fibre in combination with fluid every day [35]. Additionally, buddies will be equipped with a nutrition-related game which includes nutritional aspects.

Physical intervention

Twice a week physical training will be performed by the malnourished frail subjects together with the buddies. The exercise intervention will last about 30 minutes. Additionally, buddies will advise the malnourished (pre)frail elderly to practice the same exercise once a week on their own. The training comprises a multicomponent training with a focus on the sports motor components mobility, strength, and balance. Therefore the exercises have been selected according to the special needs of elderly people and are based on international recommendations.

- *Warm up*: Participants will be instructed to perform a 5 to 10 minute warm up with mobilization exercises.
- *Balance Exercises*: After warm up, short balance exercises will be performed.
- *Resistance Training*: In order to train the major muscle groups, 6 exercises for the lower and upper body will be accomplished.

In the first two weeks it is essential that the malnourished (pre)frail elderly learn the process of the exercises and get used to physical strain. After that, the resistance exercises will be performed with two sets and approximately 15 repetitions, which corresponds approximately the individual’s RM (repetition maximum) [36-42].

Materials:

- The pictorial guidebook will include a detailed description of all exercises with pictures of the elderlies while their physical training and the contact information of the buddies.
- For guidance and motivation all exercises will be recorded on a demonstration DVD in two levels of difficulty. Hence, both sides the malnourished (pre)frail elderly and the buddies may benefit from the training.
- Dynabands will be used to perform the resistance exercises.
- In case of questions or problems concerning the exercise program, patients have the opportunity to contact their buddies.

Course of the main study from the point of view of malnourished (pre)frail elderly subjects

1. Participants will be contacted by the trained study personnel of the MUW (Medical University Vienna) in the general hospital of Vienna (AKH), Wilhelminen hospital, hospital of Hietzing und Rudolfstiftung hospital, if they fulfill the inclusion criterias: age ≥ 65 years, living in Vienna, community dwelling, have the ability to walk, no severe impaired cognitive function, and no medical contraindication for the performance of physical strength training.
2. After they have signed the informed consent for the MNA, the SHARE-FI and the MMSE, those tests will be performed by the trained study personnel of the MUW. This will take about 20 minutes. When the subjects fulfill the criteria malnutrition or at risk of malnutrition, according to MNA OR frailty or pre-frailty according to SHARE-FI, no severe impaired cognitive function according to MMSE, and not fulfilling other exclusion criteria, they will be offered to participate in the study.
3. After discharge, the malnourished (pre)frail person will be assigned to a buddy who lives close to the malnourished (pre)frail person (Vienna north, east, south, and west) and afterwards this pairs will be assigned randomly, stratified by handgrip strength, into the intervention or the control group.
4. The included participants (buddy and malnourished (pre)frail subjects) will undergo several measurements. Muscle strength, body weight and height, BIA, quality of life, social support, frequency of hospitalization and falls, fear of falling amount of physical activity, quality of diet, quantitative and qualitative of the contents of refrigerators and demographic data will be assessed. The blood samples will be taken by the mobile services of the laboratory (IMCL – Synlab) and these samples are exclusively study-related. These laboratory values will be financed exclusively by the project. Measurements for malnourished (pre)frail subjects will be made at their home by the study personnel of the MUW and mobile services of the laboratory, respectively, and measurements for buddies will be made during the information meetings.
5. From now on they will meet twice a week. If the participant is assigned to the intervention group they will talk about nutrition and perform strength training. If the participant is assigned to the control group, assessment will take place in the same way, but the buddy will, however, not perform a specific nutrition and training intervention. After 10-12 weeks, the study participant (buddy and malnourished (pre)frail person) will again undergo several measurements. The same assessments will be carried out as at the first visit. From now on, in both groups nutrition and training intervention will be performed with the buddies twice a week.

6. After 6 months, assessment will take place again.
7. From now on, the mandatory part of the study is finished. However, it is recommended, that the visits by the buddies should continue twice a week, and both shall perform the nutrition intervention, and the strength training.
8. One year after inclusion, an evaluation is planned.

3.1 Study design

The study is designed as a prospective, randomized, controlled trial and a stratified randomization design will be performed to achieve comparable groups. It is used to ensure that the baseline variable (handgrip strength) is more evenly distributed between groups. Subjects are randomly assigned to intervention- or control group stratified by handgrip strength. Randomizer for Clinical Trials 1.8.1 (https://www.meduniwien.ac.at/randomizer/web/login.php) will be used to derive the randomization list. Each subject (malnourished (pre)frail subjects and buddies) will receive an appropriate randomization number. Participants will be assigned to their group according to their number. Randomization will be carried out after the patient has signed the informed consent form respectively after discharge, right before the baseline assessment. For analysis of the primary endpoint, all patients will be analyzed according to the intention-to-treat principle (ITT).

3.2 Major parameter

3.2.1 Primary endpoint

- *Handgrip strength*
  - Dynamometer: Hand grip strength is measured with a hand grip dynamometer in the sitting patient with elbow flexed at 90°. Subjects are asked to compress the dynamometer as strong as possible. For each side two attempts are made, and the better one is used for the calculation [43].

3.2.2 Secondary endpoints

- *Nutritional status*
  - MNA-LF: Risk for malnutrition is assessed with the long form of the mini nutritional assessment (MNA) [44]. This questionnaire consists of a screening and an assessment part. The screening part comprises 5 standardized questions concerning loss of appetite, weight loss in the last three months, mobility, actual disease/distress, psychological situation, and additionally

anthropometric measures (body mass index or calf circumference). In our study calf circumference will be used. It is measured with a tape at the sitting patient on the left and the right free pendulous lower leg at the strongest circumference. The highest value reachable in the MNA (screening part) is 14 points. Subjects with 7 points or lower were classified as malnourished, subjects with 8 to 11 points as at risk of malnutrition and 12 to 14 indicates a normal nutritional status. The assessment part of this questionnaire provides additional information about the causes of malnutrition and the highest value reachable is 16 points. Final Score (Malnutrition Indicator Score) classify subjects with 17 points or lower as malnourished, subjects with 17 to 23.5 points as at risk of malnutrition and 24 to 30 indicates a normal nutritional status [45].

- *Frailty status*
  - SHARE-FI: For the assessment of frailty the SHARE-FI as used in the Survey of Health, Ageing and Retirement in Europe [16] will be applied. This is an instrument following the main established criteria of frailty such as weight loss, exhaustion, weakness, slow walking speed, and low physical activity level [13]. The instrument consists of 4 questions regarding appetite, exhaustion, functional performance (walking 100 meter or climbing stairs), and physical activity. Furthermore, the instrument comprises hand grip strength. According to the results of the SHARE-FI, subjects are categorized sex-specifically in frail, pre-frail, and robust subjects [16].

- *Quality of life*
  - WHOQOL-BREF: Quality of life is assessed by the WHOQOL-BREF instrument. The World Health Organization Quality of Life comprises 26 items measuring the domains of physical health, psychological health, social relationships, and environment. The WHOQOL-BREF is a shorter version of the original WHO-instrument that may be more convenient for this particular study purpose [46].

  - WHOQOL-OLD: The WHOQOL-OLD is specially a project aims at developing and testing a quality of life assessment for older people. This was part of the WHOQOL - project, a collaboration of 22 research centres from different cultures under the auspices of the WHO, with the aim to develop an instrument for the measurement of subjective quality of life. For our study we

ask 3 dimensions of the six facets such as "sensory functions", "autonomy", "activities in the past, present and future" [47].

- *Social support*
    - FSozU K-14: Social support will be conducted by the short version of the Social Support Questionnaire "F-SozU" with 14 items by Fydrich et al. [48]. This questionnaire measures the emotional component of social support. Examples of the 14 items are: "I have a very close person, on whose help I can always count", and "I have friends/family members who take the time and definitely listen to me, when I need to talk". The 5-item Likert scale will be used by Eisele et al. [49]. They adapted this scale, which was suggested by Kelsey et al. [50], for the assessment of elderly patients to include yes/no answers. Further a sum score will be calculated, this ranging from 0-14 and the highest scores will indicate high level of social support [48].
- *Frequency of hospitalization*
- *Frequency of falls*
- *Fear of falling*
    - Falls Efficacy Scale International (FES-I) was developed for the documentation of fall-related self-efficacy in older persons by an EU-funded expert network (Prevention of Falls Network Europe ProFaNE). In the account the easy and more complex physical and social activities have been added [51].
- *Sings of malnutrition and frailty*
    - Laboratory parameters such as albumin, total cholesterol, transferrin, triglyceride, 25-OH-VitD3, folic acid, CRP, IL-6, TNF-Alpha and leucocytes will be measured. Blood samples will be taken from the malnourished (pre)frail subjects and the buddies by the mobile services of a laboratory company (IMCL-Synlab). Chronic inflammation is associated with both, malnutrition [7, 8] and frailty [52, 53]. Parameters of chronic inflammation like leukocytes, Interleukin 6 (IL-6), tumour necrosis factor (TNF-Alpha) [54, 55] and C reactive protein (CRP) [54-56] are associated with malnutrition and frailty. Vitamin D is essential for bone, muscle, and nerve function [29] and deficits are a sign of malnutrition [20, 57]. Further laboratory signs of malnutrition are low levels of serum proteins like albumin, transferrin, and low levels of total cholesterol and triglycerides [58].

- *Cognitive function*
  - MMSE: Measurement of cognitive function will be carried out with the German version [59] of the mini mental state examination [60]. This tool has been judged to be a screening test with satisfactory reliability and validity to assess the severity of cognitive impairment quantitatively and to document cognitive changes over time [61]. The instrument consists of 30 questions. According to the results of the MMSE, subjects are categorized in no cognitive impairments (30-25 points), slight cognitive impairments (24-18 points) and severe impaired cognitive function (≤17 points) [59].
- *Body composition*
  - BIA (Bioelectrical Impedance Analysis) is a commonly used method for estimating body composition. BIA allows the determination of fat-free mass (FFM), total body water (TBW), lean body mass (LBM), body fat (FM), body cell mass (BCM) and extracellular mass (ECM) [62]. In our study we are going to use BIA 2000-S [63]. For the measurement of the weight we are going to use the Scale Mardsen PS 250.
- *Measurement of muscle strength*
  - MASS (measurement of age and sex related reference values of muscle strength) is a new diagnosis system which was developed by the Technical University of Vienna (Dep. of Computer Engineering). It evaluates health related concentric dynamic muscle strength. The measurement is velocity-independent. Hence it is appropriate for the target group. In this study large muscle groups will be tested with this instrument. For all tested muscle groups the following parameter will be recorded: maximum resistance with low velocity; and 2-3 fix adjusted sub-maximum loads. Moreover, the Concept 2 dyno is used for assessing the muscle strength. The same exercises as before will be conducted in standardized procedure [64].
- *Amount of physical activity*
  - Physical Activity Scale for the Elderly: (PASE) is an instrument that measures the level of physical activity and to assess the effectiveness of interventions for individuals aged 65 and older. The questions capture activities in sitting situation (out of bed), walk (outside of home), and activities of different intensity (muscle training: mild, moderate, heavy exhausting). The scores are

calculated from weights and frequency values for each of the 12 types of activities. A high score indicates a high level of physical activity [65].

- *Balance and mobility components*
  - The Short Physical Performance Battery: SPPB is a method for assessing physical performance of older patients. It includes strength, balance and mobility components in one assessment tool (five chair stands, 8 feet walk test and 3 balance tests). A total SPPB Score of 0-12 is created by summing up the three components [66].
- *Qualitative and quantitative contents of refrigerator*
  - Predefined protocol of the refrigerator of the (pre)frail individuals will assess the refrigerator contents which will be classified as adequate, inadequate (rotten food or just beyond the date stamped on the label), or empty (less than three different food products) [67].
- *Quality of the whole diet*
  - MEDIAS: It is a validated 14-point Mediterranean Diet Adherence Screener used in the Prevencion con Dieta Mediterranea (PREDIMED) study for rapid estimation of adherence to the Mediterranean diet [68].
  - FFQ: Food frequency questionnaire assessing the protein intake and questions concerning supplements [69]. It will assess the usual consumption of foods during the last 2 months using standard portion sizes e.g. 1 handful per month/week/day. The questionnaire includes a list of 39 single foods and/or food categories, and the frequency of consumption is assessed according to frequency categories ranging from “never” to “several times a day”.
- *Daily physical activity*
  - Pedometer: After recruitment, each buddy receives a pedometer to measure the daily steps. Data will be stored automatically for 41 days and afterwards it has to be read out with the “Omron Health Management Software”.
- *Measures of drop-out [70]*
  - For each case of discontinued participation, the following reasons will be discriminated: medical reason (r_1), subject's own decision (r_2). Certain sets (groups) will be aggregated:
    - A – all participants who passed the baseline assessment

    - DO_i – participants who drop out by reason r_i
    - $DO_R = DO_1 + DO_2$ – all participants who drop out
    - $DO_{R1} = DO_1$ / A – participants who discontinue by medical reasons.
    - $DO_{R2} = DO_2$ / A – participants who discontinue on their own decision. Members of this group will be asked for a personal reason (voluntary and open question), and also be asked to participate in the final visit.
    - $|DO|$ – total number of participants who drop out after visit 1
    - $|DO_i|$ – number of participants who discontinue for reason R_i

- *Measures of adherence [70]*
  - Measures of adherence are calculated:
    - Number of home visits by buddies
    - Number of activity units (home exercises) and nutrition intervention units.

- *Appraisal by participant (malnourished pre/frail persons and buddies) [70]*
  - Questions will be asked such as “Would you once again participate in the trial? Why/why not?”. Participants who completed the intervention will be asked to rate the utility of the intervention program (on a scale from 1 to 5): introduction into the program, portfolio, elastic resistance bands, nutrition-related games and questionnaires. Participants will also be asked to rate the length of the program, and the frequency of home visits. Finally, an open question on concrete proposals for program improvements will be asked (“Do you have any ideas or proposals for program improvements?”)

- *Documentation of any undesirable event during the intervention [70]*
  - Any symptom or disease of any participant, which occurs during the intervention, is called an undesirable event. This definition is valid without reference to the fact, whether the intervention might be a cause or not. In case of an undesirable event, the participant promptly has to suspend nutritional and exercise units and to visit the general practitioner. The event has to be documented by a standardized report protocol, including the following judgments: medical/ non-medical; caused/not caused by exercise; subject may/may not continue nutritional and exercises units.

3.3 Statistical Analysis

All statistical analyses will be performed with IBM® SPSS® Statistics for Windows, Version 20 software (IBM Corp., Armonk, NY, U.S.). P-values < 0.05 will be considered statistically significant and all tests will be tow-sided.

Data exploration using descriptive statistical analysis and inferential statistics (uni- and multivariate) will be performed. The sample data will be carried out by frequencies or percentages (categorical variables), means and standard deviation (continuous variables), Chi-Squared-test (for categorical data) and graphics (e.g. boxplots, scatterplots and histograms). 95% confidence intervals (CI) will be calculated for the differences in percentages and medians. Testing for normal distribution will be performed by histogram, box plots.

Independent samples t-test, Mann-Whitney U test and Chi-square and Fisher-exact-test (categorial variables) of association will be used as appropriate to compare groups at baseline. Pearson's- (continuous variables) and Spearman's correlation coefficients (categorial variables) will be used.

If normal distributions are not met, non-parametric tests such as Wilcoxon, Mann-Whitney-U-Test (continuous variables) and Kendall's tau and Spearman's correlation coefficients (continuous and categorial variables) will be chosen.

- **Primary endpoint**

  Analysis of covariance, comparing handgrip strength after 10-12 weeks between intervention and control group, adjusting for the baseline value as covariate will be performed.

  Analysis by intention to treat principle: requires an assumption on the outcome value for patients in whom the planned intervention is not completed as planned in the protocol, or in whom the outcome value cannot be assessed due to hospitalization, withdrawal of consent or death.

  In all patients the 10-12 weeks handgrip strength value will be assessed, irrespective of whether the intervention was completed per protocol or not. In case that this is not possible, the baseline value will be imputed for the main analysis. A sensitivity analysis will be performed, imputing the baseline value divided by 2 for these patients.

- **Secondary endpoints**

  Analysis of covariance or Wilcoxon test will be used to compare the nutritional status (MNA, laboratory parameters), frailty status (SHARE-FI), quality of life (WHOQOL), social support (FSozU), frequency of hospitalization and falls, confidence of falls (FES-I), cognitive function (MMSE), muscle strength (MASS, concept dyno 2), body composition

(BIA), balance and mobility (SPPB) and qualitative and quantitative contents of refrigerator (protocol + picture), changes in protein intake retrieved from FFQ and adherence to Mediterranean Diet after 10-12 weeks between intervention and control group.

4. Financial Frame

The project is funded by the WWTF. It is totally feasible in terms of financial capabilities.

5. Insurance

An insurance for all study participants, the PhD-students, and the PI will be contracted via the “Koordinierungszentrum für klinische Studien” under the master policy of the Medical University of Vienna with “Zürich Versicherungs Aktiengesellschaft” (policy no. 07229622-2, ser. no. 39/2013).

6. Risk and benefits

No risk is expected for patients in the intervention group and in the control group.

The prospective benefits will have positive impact on the well-being and quality of life of the individual (micro level) as well as on the financial viability of the health system (macro level).

On an individual basis the proposed program is expected to...

- ...improve the health status of malnourished (pre)frail elderly people (decreased morbidity, decreased hospitalization and institutionalization rates, improved nutrition status, muscle strength, and quality of life)
- ...improve the health status of younger fit volunteers through their engagement with nutritional and training issues
- ...improve social interaction and social capital of both, which is also an important health resource
- ...introduce a structure which can be well implemented in long-term care of community dwelling elderly subjects with different health hazards.

The prospective benefits are also in the applicability of the project results to other regions and settings in Austrian as well as in other countries.

Measuring Project success

Micro level:

Project success will be measured on an individual basis. Therefore (pre)frail people and buddies will be tested after 10-12 weeks and 6 months as well as 1 year after project end. A positive development of the nutritional and strength parameters in malnourished (pre-)frail persons as well as in buddies will be considered as project success.

Macro level:

Track will be kept on hospitalization rates after and/or during intervention. Differences in hospitalization rates between treated and untreated subjects can be used to calculate changes in public healthcare expenses.

7. Workplan

The project is planned to have a duration of three years.

ID	Task Name
1	**Submission to the local ethics commission**
2	Protocol
3	M1 Positive ethical vote
4	**Developing phase**
5	Portfolie for control group
6	Portfolio for intervention group
7	Cooking course for Buddies
8	Training for Buddies
9	**Pilot study (Feasibility study)**
10	**Recruitment of Buddies**
11	Training of Buddies
12	Recruitment of malnourished frail persons
13	**Survey**
14	Visit 1
15	Intervention group
16	Visit 2
17	Adaptations
18	M2 Termination of pilot study
19	**Main Study**
20	**Wave 1: Recruitment of Buddies**
21	Training of Buddies
22	**Wave 2: Recruitment of Buddies**
23	Training of Buddies
24	**Wave 3: Recruitment of Buddies**
25	Training of Buddies
26	**Wave 4: Recruitment of Buddies**
27	Training of Buddies
28	Recruitment of malnourished frail persons
29	**Survey**
30	Visit 1 (several times)
31	Intervention group
32	Control group
33	Visit 2 (several times)
34	Intervention
35	Visit 3 (several times)
36	M3 Termination of main study
37	**Project completetion**
38	Statistical Analysis
39	Publication and dissemination

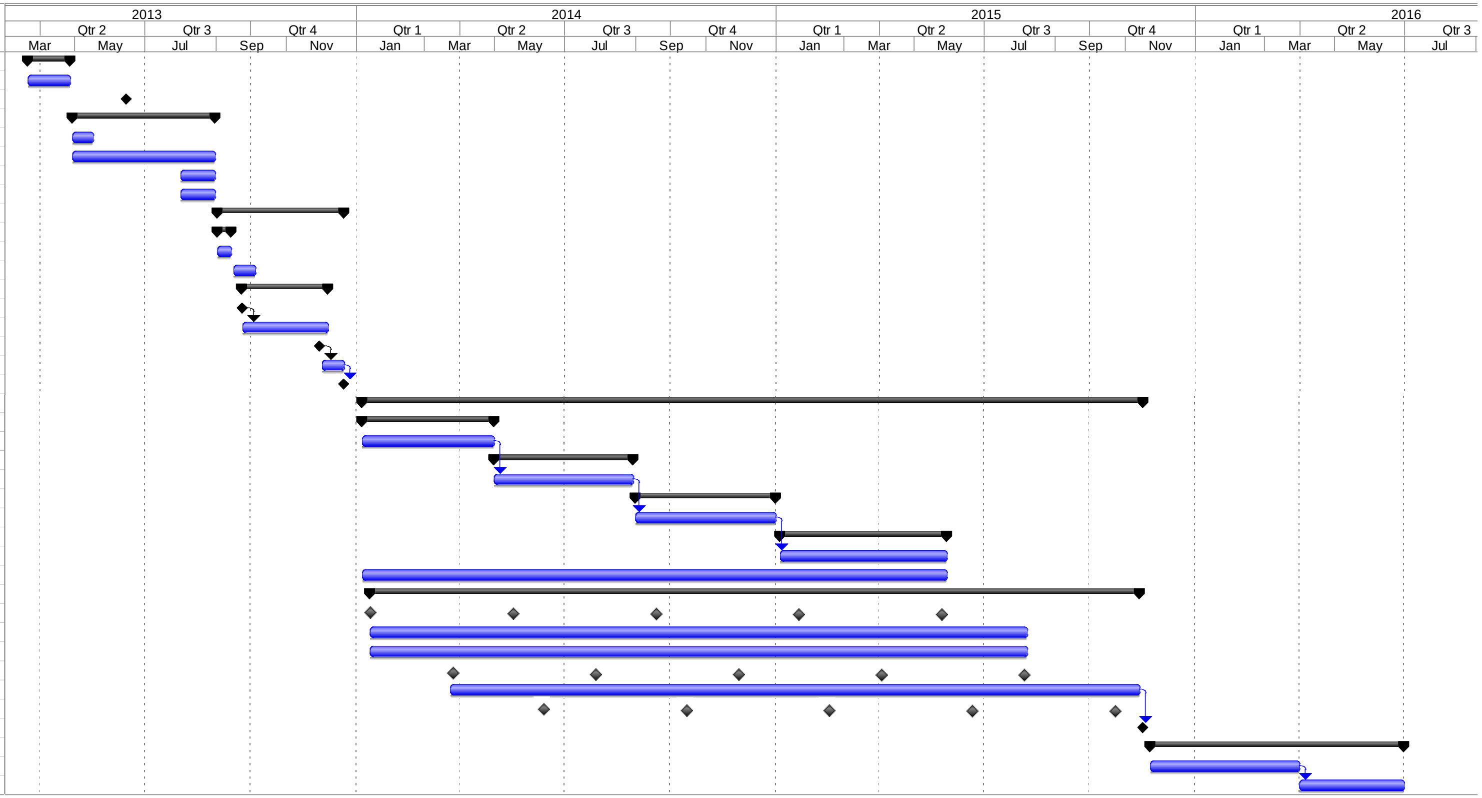

b. Phase I: Development phase

In this phase the prerequisites for the buddy visits at the malnourished frail subjects will be developed. Those include a portfolio with photographs and recipes for protein rich food, a video with exercises with two different difficulty levels and as alternative for those without video / DVD recorder a portfolio with pictures of exercises will be designed. Further, nutrition-related game will be developed. In this phase instruction material (compendium) for buddies will also be developed. A portfolio for the control-group will be developed with memory- and concentration games.

c. Phase II: Pilot study (Feasibility study)

In this phase the first 10 buddies will be instructed, trained and matched with 10 malnourished, (pre)frail elderly subjects, who will be also recruited in this phase. All parameters that will be assessed in the main study phase will be applied in this pilot phase. For the pilot phase no control group is planned.

d. Phase III: Main study phase

In this phase malnourished, (pre)frail elderly subjects and buddies will be randomized in two groups, an intervention and a control group. Participants in the control group will be visited regularly by buddies, but no specific nutrition training or physical exercises will take place within the 10-12 weeks. After this first phase of 10-12 weeks, nutrition and strength training intervention will be performed also with the control group.

Buddies will be recruited in 4 waves, in each phase 30 buddies.

The follow-up consists of three contacts, conducted approximately one week after discharge, after 10-12 weeks and after 6 months. Each couple (one buddy and one malnourished (pre)frail elderly subjects) will be assessed three times, independent of the group they are in. After 6 months the compulsory part of the buddy-study person couple ends, but they will be encouraged to keep on their visits and engage in strength training. Each couple will be evaluated again 1 year after their beginning according to continuity of the visits and quality of life.

Timetable:

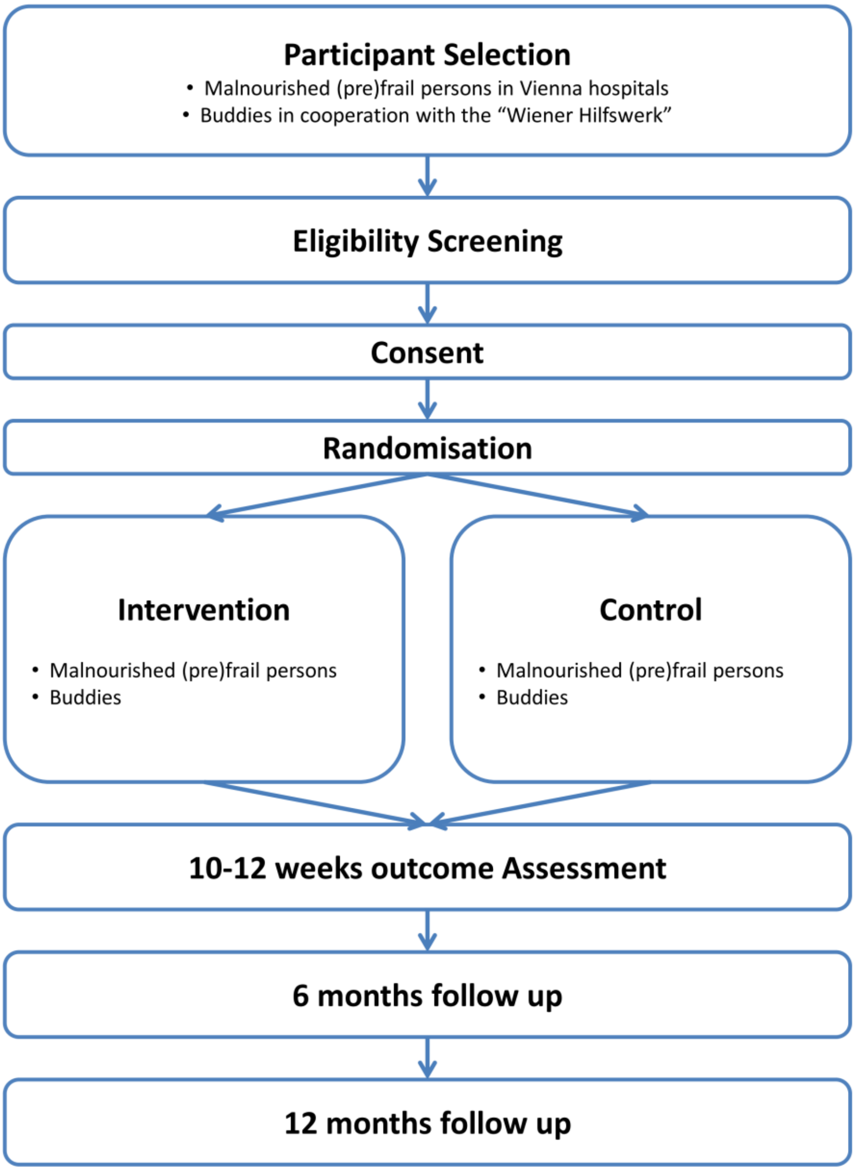

8. Regulatory framework

8.1 Data administration

All patients will be coded anonymous. Patients' individual data will be anonymized entered in a secure web authentication online questionnaire which will be programmed for this trial. Patient's data will be encrypted with a Secure Sockets Layer (SSL) RSA 1024 bit certificate.

The data will be entered in SPSS data file only with this code. Only authorized persons have access to the original data.

8.2 Ethical and legal aspects

The study will be performed according to the Helsinki declaration (1964). The protocol will be submitted for review at the Ethical committee of the Vienna Medical University. Patients have to sign the written informed consent before study participation.

8.3 Patients' informed consent

Patients will be informed orally about the purpose, the course of the study the associated examinations and the possible consequences. Informed consent has to be signed before study entry. Patients will receive a copy of the informed consent.

8.4 Documentation of the study results

All findings and results will be documented in a case report file (CRF). Corrections of data will be performed with clearly indicating who has modified data.

8.5 Intended use of the data

Patients' individual data will be anonymized entered in a SPSS data file. Patients' individual data will be accessible for the study team. Individual patients' data will not be traceable and identifiable in the database.

The study results will be published by the study team in scientific journals as well as presented during scientific conferences.

8.6 Protocol changes and amendments

Any change of the protocol will be submitted to the ethical committee as protocol amendment and has to be approved by the ethical committee.

9. References

1. Allison, S.P., Malnutrition, disease, and outcome. Nutrition. 2000; 16(7-8): p. 590-3.
2. Davison, K.K., et al., Percentage of body fat and body mass index are associated with mobility limitations in people aged 70 and older from NHANES III. J Am Geriatr Soc. 2002; 50(11): p. 1802-9.
3. Dorner, T.E. and A. Rieder, Obesity paradox in elderly patients with cardiovascular diseases. Int J Cardiol. 2012; 155(1): p. 56-65.
4. Dorner, T.E., et al., Body mass index and the risk of infections in institutionalised geriatric patients. Br J Nutr. 2010; 103(12): p. 1830-5.
5. Locher, J.L., et al., Body mass index, weight loss, and mortality in community-dwelling older adults. J Gerontol A Biol Sci Med Sci. 2007; 62(12): p. 1389-92.
6. Zizza, C.A., et al., Obesity affects nursing-care facility admission among whites but not blacks. Obes Res. 2002; 10(8): p. 816-23.
7. Soeters, P.B., et al., A rational approach to nutritional assessment. Clin Nutr. 2008; 27(5): p. 706-16.
8. Soeters, P.B. and A.M. Schols, Advances in understanding and assessing malnutrition. Curr Opin Clin Nutr Metab Care. 2009; 12(5): p. 487-94.
9. Blaum, C.S., et al., The association between obesity and the frailty syndrome in older women: the Women's Health and Aging Studies. J Am Geriatr Soc. 2005; 53(6): p. 927-34.
10. Espinoza, S.E. and L.P. Fried, Risk Factors for Frailty in the Older Adult. Clin Geriatrics. 2007; 15: p. 37-44.
11. Villareal, D.T., et al., Physical frailty and body composition in obese elderly men and women. Obes Res. 2004; 12(6): p. 913-20.
12. Woods, N.F., et al., Frailty: emergence and consequences in women aged 65 and older in the Women's Health Initiative Observational Study. J Am Geriatr Soc. 2005; 53(8): p. 1321-30.
13. Fried, L.P., et al., Frailty in older adults: evidence for a phenotype. J Gerontol A Biol Sci Med Sci. 2001; 56(3): p. M146-56.
14. Fried, L.P., et al., Untangling the concepts of disability, frailty, and comorbidity: implications for improved targeting and care. J Gerontol A Biol Sci Med Sci. 2004; 59(3): p. 255-63.
15. Espinoza, S. and J.D. Walston, Frailty in older adults: insights and interventions. Cleve Clin J Med. 2005; 72(12): p. 1105-12.
16. Romero-Ortuno, R., et al., A frailty instrument for primary care: findings from the Survey of Health, Ageing and Retirement in Europe (SHARE). BMC Geriatr. 2010; 10: p. 57.
17. Santos-Eggimann, B., et al., Prevalence of frailty in middle-aged and older community-dwelling Europeans living in 10 countries. J Gerontol A Biol Sci Med Sci. 2009; 64(6): p. 675-81.

18. Böck, M., A. Rieder, and T.E. Dorner, *Frailty. Definition, Erkennung und Bedeutung in der Gesundheitsförderung und Prävention.*, in *Gesundheitswissenschaften*. 2011: Linz.
19. Bartali, B., et al., Low nutrient intake is an essential component of frailty in older persons. J Gerontol A Biol Sci Med Sci. 2006; 61(6): p. 589-93.
20. Ble, A., et al., Lower plasma vitamin E levels are associated with the frailty syndrome: the InCHIANTI study. J Gerontol A Biol Sci Med Sci. 2006; 61(3): p. 278-83.
21. Michelon, E., et al., Vitamin and carotenoid status in older women: associations with the frailty syndrome. J Gerontol A Biol Sci Med Sci. 2006; 61(6): p. 600-7.
22. Elmadfa, I., et al., *Österreichischer Ernährungsbericht 2008*. 2009: Wien, März.
23. Dorner, T.E., et al., Prevalence of obesity in a geriatric long-term care facility. JNHA 2006; 10(72).
24. Bollwein, J., et al., Dietary Quality Is Related to Frailty in Community-Dwelling Older Adults. J Gerontol A Biol Sci Med Sci. 2012; [Epub ahead of print].
25. Dorner, T., et al., The effect of structured strength and balance training on cognitive function in frail, cognitive impaired elderly long-term care residents. Aging Clin Exp Res. 2007; 19(5): p. 400-5.
26. Perrig-Chiello, P., et al., The effects of resistance training on well-being and memory in elderly volunteers. Age Ageing. 1998; 27(4): p. 469-75.
27. Vestergaard, S., C. Kronborg, and L. Puggaard, Home-based video exercise intervention for community-dwelling frail older women: a randomized controlled trial. Aging Clin Exp Res. 2008; 20(5): p. 479-86.
28. Walston, J., et al., Frailty and activation of the inflammation and coagulation systems with and without clinical comorbidities: results from the Cardiovascular Health Study. Arch Intern Med. 2002; 162(20): p. 2333-41.
29. Walston, J.D. Frailty. UpToDate. last updated: Jan 5, 2012. http://www.uptodate.com/contents/frailty.
30. Dorner, T.E., J. Tschinderle, and K. Schindler, Frailty, Ernährungsstatus und Bereitschaft zu einer Trainings- und Ernährungsintervention bei älteren Personen in Wiener Krankenhäusern. Wien Klin Wochenschr. 2011; 123(17-18): p. A40-A41.
31. Tschinderle, J., *Frailty, Ernährungsstatus und Bereitschaft zu einer Trainings- und Ernährungsintervention bei älteren und hochbetagten Personen über 65 Jahre in Wiener Krankenhäusern. Masterarbeit*. Universität Wien, Wintersemester 2011/12.
32. Tschinderle, J., K. Schindler, and T. Dorner. Prävalenz von Frailty und Malnutrition bei Personen über 65 Jahren in Wiener Krankenhäusern. last updated: 16.04.2012. http://www.pflegenetz.at/index.php?id=82&tx_ttnews%5Btt_news%5D=527&cHash=fc936f9e463b339fc487cd6d1315b4b8&print=1.
33. Robinson, S.M., et al., Diet and its relationship with grip strength in community-dwelling older men and women: the Hertfordshire cohort study. J Am Geriatr Soc. 2008; 56(1): p. 84-90.
34. Seco, J., et al., A long-term physical activity training program increases strength and flexibility, and improves balance in older adults. Rehabil Nurs. 2013; 38(1): p. 37-47.

35. Rydwik, E., K. Frandin, and G. Akner, Effects of a physical training and nutritional intervention program in frail elderly people regarding habitual physical activity level and activities of daily living--a randomized controlled pilot study. Arch Gerontol Geriatr. 2010; 51(3): p. 283-9.

36. Hauer, K., et al., Exercise training for rehabilitation and secondary prevention of falls in geriatric patients with a history of injurious falls. J Am Geriatr Soc. 2001; 49(1): p. 10-20.

37. Alexander, N.B., et al., Task-specific resistance training to improve the ability of activities of daily living-impaired older adults to rise from a bed and from a chair. J Am Geriatr Soc. 2001; 49(11): p. 1418-27.

38. Bean, J.F., et al., Increased Velocity Exercise Specific to Task (InVEST) training: a pilot study exploring effects on leg power, balance, and mobility in community-dwelling older women. J Am Geriatr Soc. 2004; 52(5): p. 799-804.

39. Fiatarone, M.A., et al., Exercise training and nutritional supplementation for physical frailty in very elderly people. N Engl J Med. 1994; 330(25): p. 1769-75.

40. Yarasheski, K.E., et al., Resistance exercise training increases mixed muscle protein synthesis rate in frail women and men >/=76 yr old. Am J Physiol. 1999; 277(1 Pt 1): p. E118-25.

41. Sullivan, D.H., et al., Effects of muscle strength training and megestrol acetate on strength, muscle mass, and function in frail older people. J Am Geriatr Soc. 2007; 55(1): p. 20-8.

42. Brown, M., et al., Low-intensity exercise as a modifier of physical frailty in older adults. Archives of physical medicine and rehabilitation. 2000; 81(7): p. 960-5.

43. Roberts, H.C., et al., A review of the measurement of grip strength in clinical and epidemiological studies: towards a standardised approach. Age Ageing. 2011; 40(4): p. 423-9.

44. Kondrup, J., et al., ESPEN guidelines for nutrition screening 2002. Clin Nutr. 2003; 22(4): p. 415-21.

45. Vellas, B., et al., Overview of the MNA-Its history and challenges. J Nutr Health Aging. 2006; 10(6): p. 456-463.

46. Group, T., Development of the World Health Organization WHOQOL-BREF quality of life assessment. The WHOQOL Group. Psychol Med. 1998; 28(3): p. 551-8.

47. Winkler, I., H. Matschinger, and M.C. Angermeyer, The WHOQOL-OLD. Psychother Psychosom Med Psychol. 2006; 56(2): p. 63-9.

48. Fydrich, T., G. Sommer, and E. Brähler, Fragebogen zur Sozialen Unterstützung (F-SozU). Manual. 2007; Göttingen: Hogrefe.

49. hadn, et al., Influence of social support on cognitive change and mortality in old age: results from the prospective multicentre cohort study AgeCoDe. BMC Geriatr. 2012; 12: p. 9.

50. Kelsey, J.L., et al., Issues in carrying out epidemiologic research in the elderly. Am J Epidemiol. 1989; 130(5): p. 857-66.

51. Dias, N., et al., The German version of the Falls Efficacy Scale-International Version (FES-I). Z Gerontol Geriatr. 2006; 39(4): p. 297-300.
52. Hubbard, R.E. and K.W. Woodhouse, Frailty, inflammation and the elderly. Biogerontology. 2010; 11(5): p. 635-41.
53. Kanapuru, B. and W.B. Ershler, Inflammation, coagulation, and the pathway to frailty. Am J Med. 2009; 122(7): p. 605-13.
54. Hubbard, R.E., et al., Plasma esterases and inflammation in ageing and frailty. Eur J Clin Pharmacol. 2008; 64(9): p. 895-900.
55. Leng, S.X., et al., Inflammation and frailty in older women. J Am Geriatr Soc. 2007; 55(6): p. 864-71.
56. Puts, M.T., et al., Endocrine and inflammatory markers as predictors of frailty. Clin Endocrinol (Oxf). 2005; 63(4): p. 403-11.
57. Semba, R.D., et al., Low serum micronutrient concentrations predict frailty among older women living in the community. J Gerontol A Biol Sci Med Sci. 2006; 61(6): p. 594-9.
58. Harris, D. and N. Haboubi, Malnutrition screening in the elderly population. J R Soc Med. 2005; 98(9): p. 411-4.
59. Kessler, J., P. Denzler, and H.J. Markowitsch, *Mini Mental Status Examination MMSE. German Version*, in *DT Demenz-Test: Eine Testbatterie zur Erfassung kognitiver Beeinträchtigungen im Alter*. 1990, Beltz-Verlag: Weinheim.
60. Folstein, M.F., S.E. Folstein, and P.R. McHugh, "Mini-mental state". A practical method for grading the cognitive state of patients for the clinician. J Psychiatr Res. 1975; 12(3): p. 189-98.
61. Tombaugh, T.N. and N.J. McIntyre, The mini-mental state examination: a comprehensive review. J Am Geriatr Soc. 1992; 40(9): p. 922-35.
62. Kyle, U.G., et al., Bioelectrical impedance analysis--part I: review of principles and methods. Clin Nutr. 2004; 23(5): p. 1226-43.
63. Data Imput GmbH, *Gebrauchsanleitung Jubiläumssonderserie BIA 2000 - S Bioelectrical Impedance Analyzer*. 2012.
64. Marchart, P., *Anthropometrisch- und altersbezogene Referenzwerte für die Maximalkraft und Kraftausdauer bei Kindern (ab 12 J.), Jugendlichen und Erwachsenen* 2002, University of Vienna: Vienna.
65. Washburn, R.A., et al., The Physical Activity Scale for the Elderly (PASE): development and evaluation. J Clin Epidemiol. 1993; 46(2): p. 153-162.
66. Guralnik, J.M., et al., A short physical performance battery assessing lower extremity function: association with self-reported disability and prediction of mortality and nursing home admission. J Gerontol. 1994; 49(2): p. M85-94.
67. Boumendjel, N., et al., Refrigerator content and hospital admission in old people. The Lancet. 2000; 356(9229): p. 563.
68. Schroder, H., et al., A short screener is valid for assessing Mediterranean diet adherence among older Spanish men and women. J Nutr. 2011; 141(6): p. 1140-5.

69. Kroke, A., et al., Validation of a self-administered food-frequency questionnaire administered in the European Prospective Investigation into Cancer and Nutrition (EPIC) Study: comparison of energy, protein, and macronutrient intakes estimated with the doubly labeled water, urinary nitrogen, and repeated 24-h dietary recall methods. Am J Clin Nutr. 1999; 70(4): p. 439-47.

70. Hinrichs, T., et al., Feasibility of a multidimensional home-based exercise programme for the elderly with structured support given by the general practitioner's surgery: study protocol of a single arm trial preparing an RCT [ISRCTN58562962]. BMC Geriatr. 2009; 9: p. 37.
